# Thermally Stable Capacitive Energy-Density and Colossal Electrocaloric and Pyroelectric Effects of Sm-Doped Pb(Mg_1/3_Nb_2/3_)O_3_–PbTiO_3_ Thin Films

**DOI:** 10.1021/jacs.4c11555

**Published:** 2024-11-18

**Authors:** Zouhair Hanani, Jamal Belhadi, Urška Trstenjak, Nick A. Shepelin, Vid Bobnar, Hana Uršič, Nina Daneu, Nikola Novak, David Fabijan, Anna Razumnaya, Yuri Tikhonov, Thomas Lippert, Zdravko Kutnjak, Gertjan Koster, Igor Lukyanchuk, Matjaž Spreitzer

**Affiliations:** † Advanced Materials Department, Jožef Stefan Institute, Jamova Cesta 39, Ljubljana 1000, Slovenia; ‡ Laboratory of Physics of Condensed Mater, University of Picardie Jules Verne, 33 Rue Saint-Leu, Amiens 80039, France; § Laboratory for Multiscale Materials Experiments, Paul Scherrer Institute, Forschungsstrasse 111, Villigen PSI 5232, Switzerland; ∥ Condensed Matter Physics Department, Jožef Stefan Institute, Jamova Cesta 39, Ljubljana 1000, Slovenia; ⊥ Electronic Ceramics Department, Jožef Stefan Institute, Jamova Cesta 39, Ljubljana 1000, Slovenia; # Department of Chemistry and Applied Biosciences, ETH Zürich, Zürich 8093, Switzerland; ¶ MESA+ Institute for Nanotechnology, University of Twente, AE Enschede 7500, The Netherlands

## Abstract

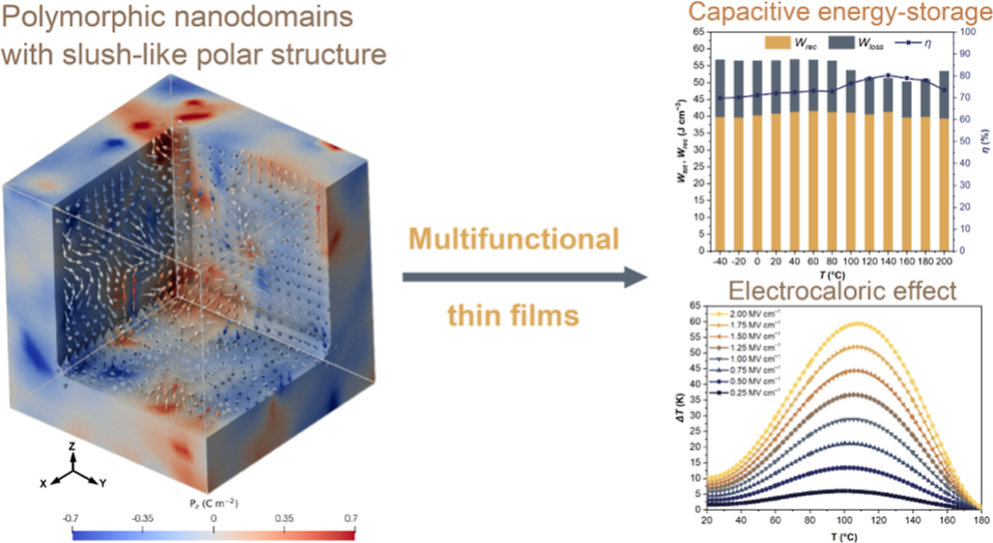

Sm-doped Pb(Mg_1/3_Nb_2/3_)O_3_–PbTiO_3_ (Sm-PMN–PT) bulk materials have revealed outstanding ferroelectric and piezoelectric properties due to enhanced local structural heterogeneity. In this study, we further explore the potential of Sm-PMN–PT by fabricating epitaxial thin films by pulsed laser deposition, revealing that Sm doping significantly improves the capacitive energy-storage, piezoelectric, electrocaloric, and pyroelectric properties of PMN–PT thin films. These Sm-PMN–PT thin films exhibit fatigue-free performance up to 10^9^ charge–discharge cycles and maintain thermal stability across a wide temperature range from −40 to 200 °C. Notably, the films demonstrate a colossal electrocaloric effect with a temperature change of 59.4 K and a remarkable pyroelectric energy density reaching 40 J cm^–3^. By using scanning transmission electron microscopy and phase-field modeling, we revealed that these exceptional properties arise from the increased local structural heterogeneity and strong local electric fields along spontaneous polarization directions, facilitating the nucleation of polymorphic nanodomains characterized by a slush-like polar structure. These findings highlight the enormous potential of Sm-PMN–PT films in capacitive energy storage and solid-state electrothermal energy interconversion. Furthermore, this approach holds broad potential for other relaxor ferroelectrics by enabling the manipulation of nanodomain structures, paving the way for developing robust multifunctional materials.

## Introduction

The growing demand for miniaturized and nanoscale electrical devices requires multifunctional materials capable of storing energy, rapidly releasing the stored energy, and harvesting the heat generated during energy production.^1,2^ Relaxor ferroelectrics (RFEs) have shown great promise due to their intrinsic polarization and the unique interplay between their electrical, mechanical, and thermal properties.^3^ Due to their fast electrostatic effect determined by the applied electric field (*E*) and the consequent polarization (*P*), RFEs materials can store charge and release it upon application and withdrawal of *E*.^4^ Besides, the coupling of polarization and temperature can be leveraged to harvest waste heat for clean electrical energy generation through pyroelectric energy conversion and for advanced solid-state cooling utilizing the electrocaloric effect (ECE).^5,6^ The ECE relies on applying an electric field to a polar dielectric, which causes a change in the temperature of the material.^6^ On the other hand, pyroelectric energy conversion requires thermodynamic cycles that leverage temporal variations of the thermal profiles to extract electrical work from the material,^5^ and the Olsen cycle was reported to extract the maximum potential work from a pyroelectric material.^7,8^ Furthermore, the use of thin film devices will undoubtedly provide added advantages like low thermal mass, increased breakdown strength (BDS), and enhanced susceptibilities of the functionalities mentioned above.^9^ Besides, the ability of the RFE thin film to withstand high electric field at high temperatures is crucial in intensifying the electrocaloric and pyroelectric effects.

A simple strategy for improving the BDS and mitigate the thermal breakdown is by introducing additional compositional disorder, which promotes the relaxor-like behavior.^10,11^ Particularly, the doping of Sm^3+^ into the A-site of a perovskite ABO_3_ lattice can induce strong local chemical and structural heterogeneity.^12^ These features augment the BDS by disrupting the ferroelectric order, scaling down the nanodomain size,^10^ and strengthening the thermal breakdown by depressing the thermal activation of carriers, thus reducing the conduction loss at increased temperatures.^11^ Mostly, the model of polar nanoregions (PNRs) inside a nonpolar matrix (e.g., SrTiO_3_) has been widely used to explain the enhanced energy storage performances in RFEs.^11,13,14^ Difficulties arise when attempting to explain the unusual properties of certain complex RFEs like Pb(Mg_1/3_Nb_2/3_)O_3_–*x*PbTiO_3_ (PMN–*x*PT) materials. These RFEs are characterized by the presence of a multidomain state [polar nanodomains (PNDs)] with extremely small domain sizes (2–10 nm), and the absence of a nonpolar matrix, presenting a slush-like polar structure.^15^

**Figure 1:**
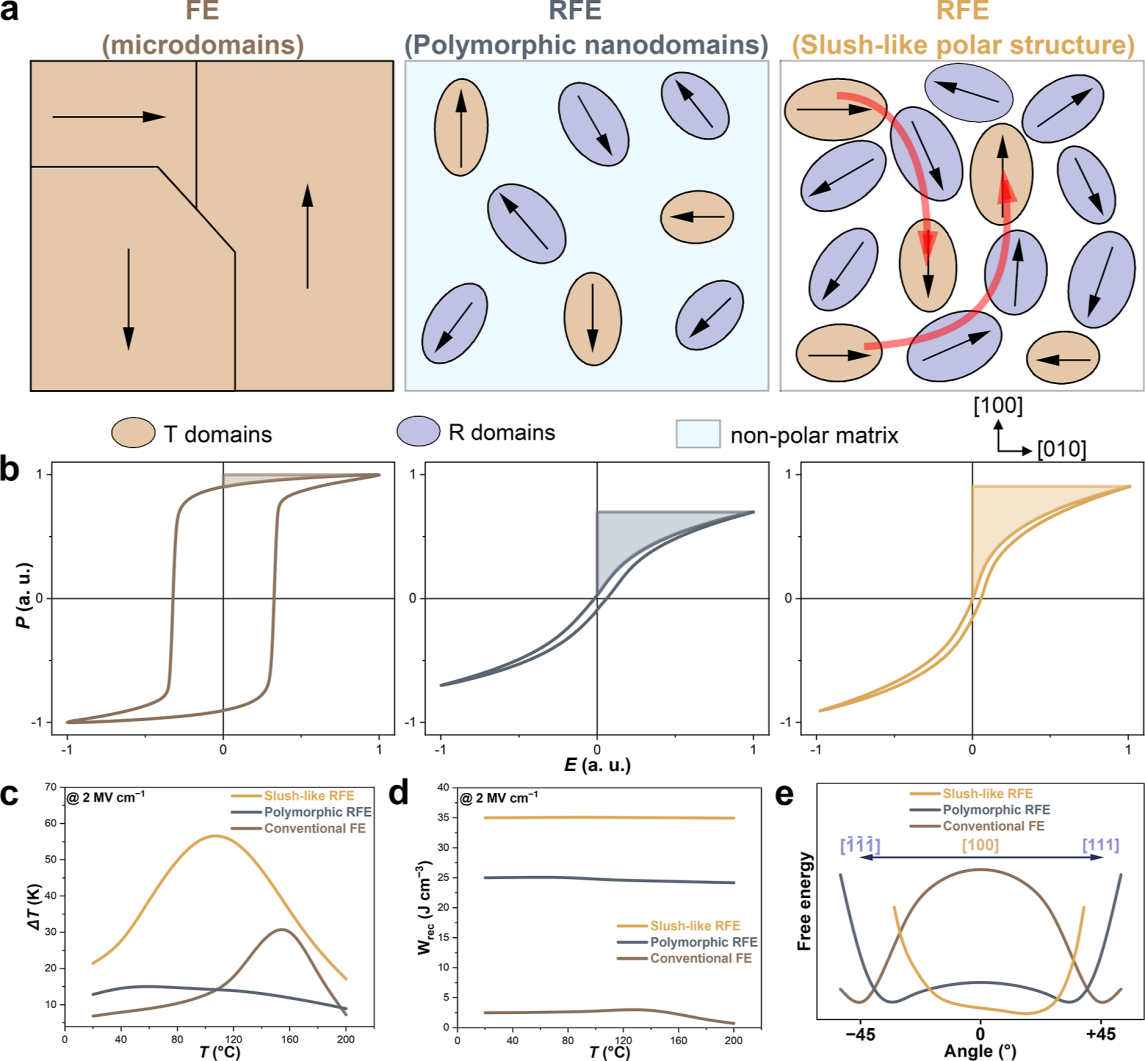
Design of multifunctional RFEs with slush-like polar structure exhibiting colossal energy storage and electrocaloric properties. (a) Comparative display domain structure and *P*–*E* loops of a conventional FE, RFE with polymorphic nanodomains and nonpolar matrix, and RFE with slush-like polar structure. The red arrows present the bridging mechanism in the slush-like RFE. (b) The corresponding *P*–*E* hysteresis loops, where the shadowed area in the *P*–*E* loops represents the energy density. Thermal evolution of the (c) capacitive energy density and (d) the adiabatic temperature change (electrocaloric effect). (e) Comparative display of Landau free energy profiles in the three-domain structures showing the two-dimensional (2D) energy profiles for a [1̅1̅1̅]–[100]–[111] polarization rotation path (*R*–*T*–*R*), where the *x*-axis represents the angle between the polar vector and the [100] direction, and the *y*-axis represents the minimal energy for a specific polar direction.

In conventional FE, where sizable ferroelectric microdomains exist (Figure 1a), their high *P* generally led to large adiabatic temperature change (Δ*T*), manifesting as the electrocaloric effect, particularly around the ferroelectric-paraelectric phase transition (Figure 1c). Unfortunately, their square-like *P*–*E* loop (Figure 1b) results in poor capacitive energy storage density (*W*_rec_) (Figure 1d). In contrast, RFE with polymorphic nanodomains, i.e., the coexistence of rhombohedral (*R*) and tetragonal (*T*) nanodomains in a nonpolar matrix (Figure 1a), exhibit enhanced *W*_rec_ due to the nonsaturated and slim hysteresis loop (Figure 1d), allowing the application of large *E* (Figure 1b). This improvement is at the expense of Δ*T* resulting from sacrificed *P* due to the nonpolar matrix (Figure 1c). In this work, we propose a multifunctional RFE exhibiting a slush-like polar structure with interconnected nanodomains exhibiting, simultaneously, colossal energy storage and electrocaloric properties (Figure 1c,d). According to the Landau phenomenological theory, the thermodynamic energy profile of an RFE with polymorphic domains should be flatter than that of a conventional FE with only *R* or *T* domains.^11^ Besides, an RFE with a slush-like polar structure and connected nanodomains can significantly reduce the domain wall energy by lowering the angles of the domain walls,^16^ leading to an even flatter energy landscape (Figure 1e). Furthermore, the presence of an internal electric field, i.e., a positive imprint of *P*–*E* hysteresis loop due to defect dipoles, for instance, will result in an asymmetric Landau energy profile, leading to improved energy density (Figure 1e). The data in Figure 1 were obtained based on previous studies.^4,10,11,17−20^

## Results and Discussions

### Capacitive Energy Storage Properties

**Figure 2:**
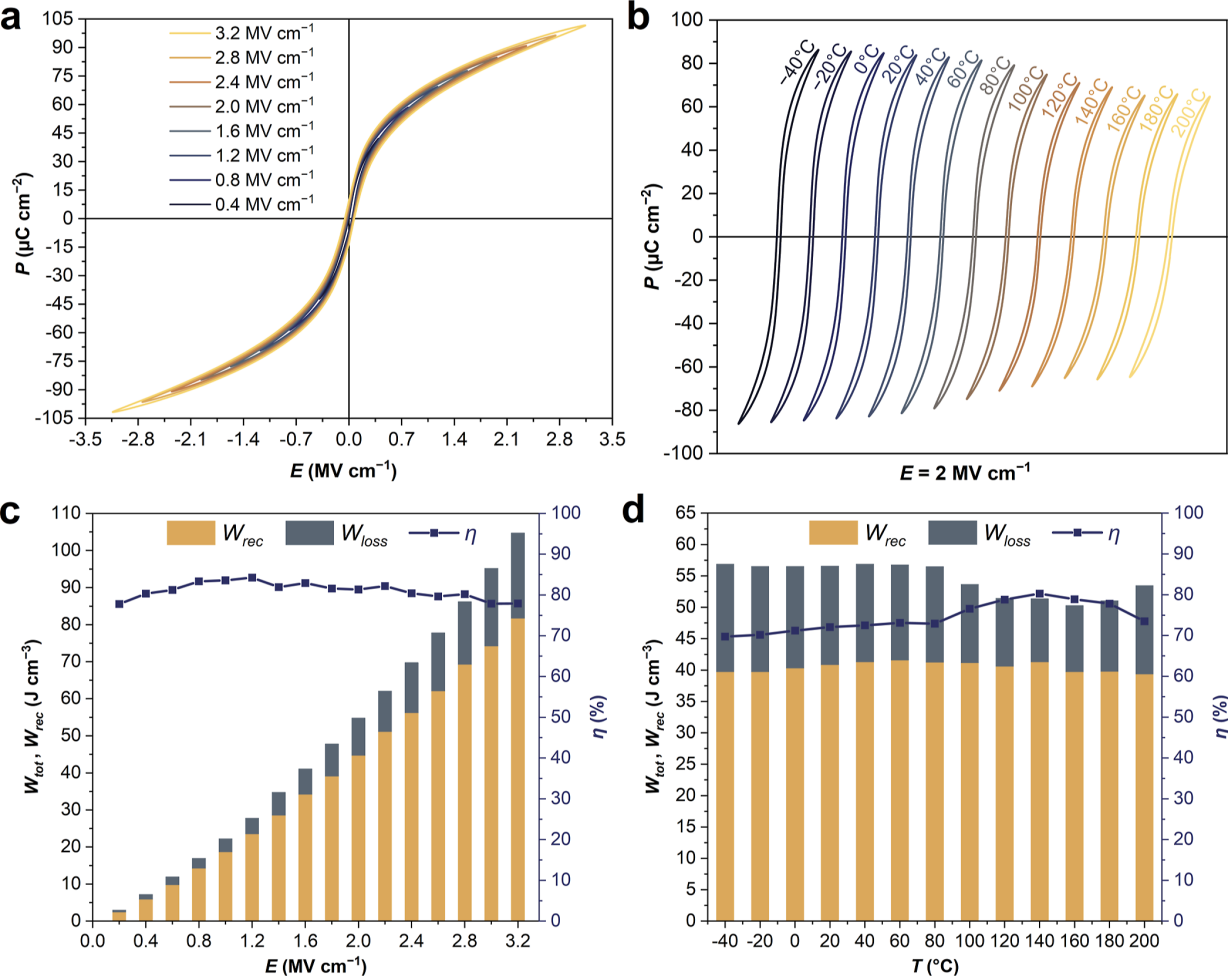
Energy storage properties of Sm-PMN–30PT film. (a) Room-temperature *P*–*E* hysteresis loops, (b) temperature dependence of the *P*–*E* loops at 2 MV cm^–1^, and (c,d) the corresponding energy storage parameters of Sm-PMN–30PT film.

In order to evaluate the capacitive energy storage properties of RFE with a slush-like polar structure, we prepared 250 nm thick PMN–30PT and 2 at. % Sm-doped PMN–30PT thin films by pulsed laser deposition (PLD). Both thin films were highly crystalline, single-phase, fully epitaxial (00*l*)-oriented, and relaxed (Figure S1). The energy storage properties of the films were derived from the *P*–*E* measurements at room temperature (Figure S5a). Both samples displayed typical characteristics of ergodic RFEs, as evidenced by nonsaturated slim hysteresis loops, high maximum polarization (*P*_max_) and low remnant polarization (*P*_r_). Notably, the positively imprinted *P*–*E* loop in Sm-PMN–30PT is highly desirable for capacitive energy storage, as a larger amount of energy can be stored.^4^ At 1 MV cm^–1^, the recoverable energy density (*W*_rec_) and the energy efficiency (η) reached (10.7 J cm^–3^, 68.7%) and (18.6 J cm^–3^, 83.6%) in PMN–30PT and Sm-PMN–30PT films, respectively. In the following sections, the study will focus solely on the Sm-PMN–30PT film due to its superior performance and potential for advanced applications. *P*–*E* hysteresis loops under various electric fields were recorded at room temperature (Figure 2a). At 3.2 MV cm^–1^, *P*_max_ and *P*_r_ reached 102.2 and 9.6 μC cm^–2^, respectively, corresponding to ultrahigh polarization change (*P*_max_ – *P*_r_) of 92.6 μC cm^–2^, leading to high energy density. We calculated the energy performance of Sm-PMN–30PT film from the field dependence of the *P*–*E* loops. Both the total energy storage density (*W*_tot_) and *W*_rec_ show a parabolic growth trend as the applied electric field increases from 0.2 to 3.2 MV cm^–1^ (Figure 2c). Consequently, a high *W*_rec_ of 82 J cm^–3^ and η of 78% are simultaneously achieved, which exceeds the values reported for PMN–PT films.^4,10,11,21−23^ For instance, at 3.2 MV cm^–1^, the obtained *W*_rec_ of our sample is higher compared to the energy storage values obtained, at the same *E*, in the ion-bombarded PMN–32PT film (*W*_rec_ = 69.2 J cm^–3^),^4^ in 0.25BiFeO_3_–0.30BaTiO_3_–0.45SrTiO_3_ RFE with polymorphic domains (*W*_rec_ = 54.8 J cm^–3^),^11^ and in the superparaelectric RFE 30 mol % Sm-doped 0.3BiFeO_3_–0.7BaTiO_3_ film (*W*_rec_ = 59.3 J cm^–3^).^10^ Besides, the breakdown strength of Sm-PMN–30PT was obtained from Weibull distribution analysis (Figure S8), where the statistical breakdown strength (*E*_b_) and Weibull modulus β (reliability indicator) are found to be 3.9 MV cm^–1^ and of 13.3, respectively.

To ensure the wide applicability of capacitors, excellent frequency, cycling and temperature stability of the energy storage performance is necessary. The Sm-PMN–30PT film has demonstrated reliable energy storage parameters across various frequencies (*W*_rec_ ≈ 12.2 ± 0.4 J cm^–3^, η ≈ 83.9 ± 2.7%) (Figure S9c) and endured one billion charge–discharge cycles while keeping constant energy storage properties (*W*_rec_ ≈ 18.6 ± 0.1 J cm^–3^, η ≈ 84.1 ± 0.2%) (Figure S9d). Furthermore, we studied the thermal stability of the energy storage performance of Sm-PMN–30PT film by recording *P*–*E* loops at various temperatures (Figure 2b), and plotted the temperature-dependent energy storage parameters (Figure 2d). A continuous decrease of the polarization and narrowing of the *P*–*E* loops from −40 to 160 °C was observed due to the ferroelectric-relaxor phase transition, and a reversed trend occurs at higher temperatures due to the thermally stimulated conduction loss.^10^ The Sm-PMN–30PT film exhibited thermally stable energy storage characteristics between −40 and 200 °C (*W*_rec_ ≈ 40.4 ± 0.8 J cm^–3^, η ≈ 74.4 ± 3.6%) at large electric field of 2 MV cm^–1^. The energy storage variation (ESV) is less than 5% between −40 and 200 °C, which is two times lower than the ion-bombarded PMN–32PT film (ESV ≈ 10.1% between −100 and 200 °C),^4^ and inferior than the 30 mol % Sm-doped 0.3BiFeO_3_–0.7BaTiO_3_ film (ESV ≈ 6% between −100 and 150 °C),^10^ at the same electric field of 2 MV cm^–1^. These results make the Sm-PMN–30PT film very suitable for capacitive energy storage at a high-temperature operation like in hybrid electric vehicles (140 °C).^11^

### Electrocaloric Effect and Pyroelectric Energy Harvesting

**Figure 3:**
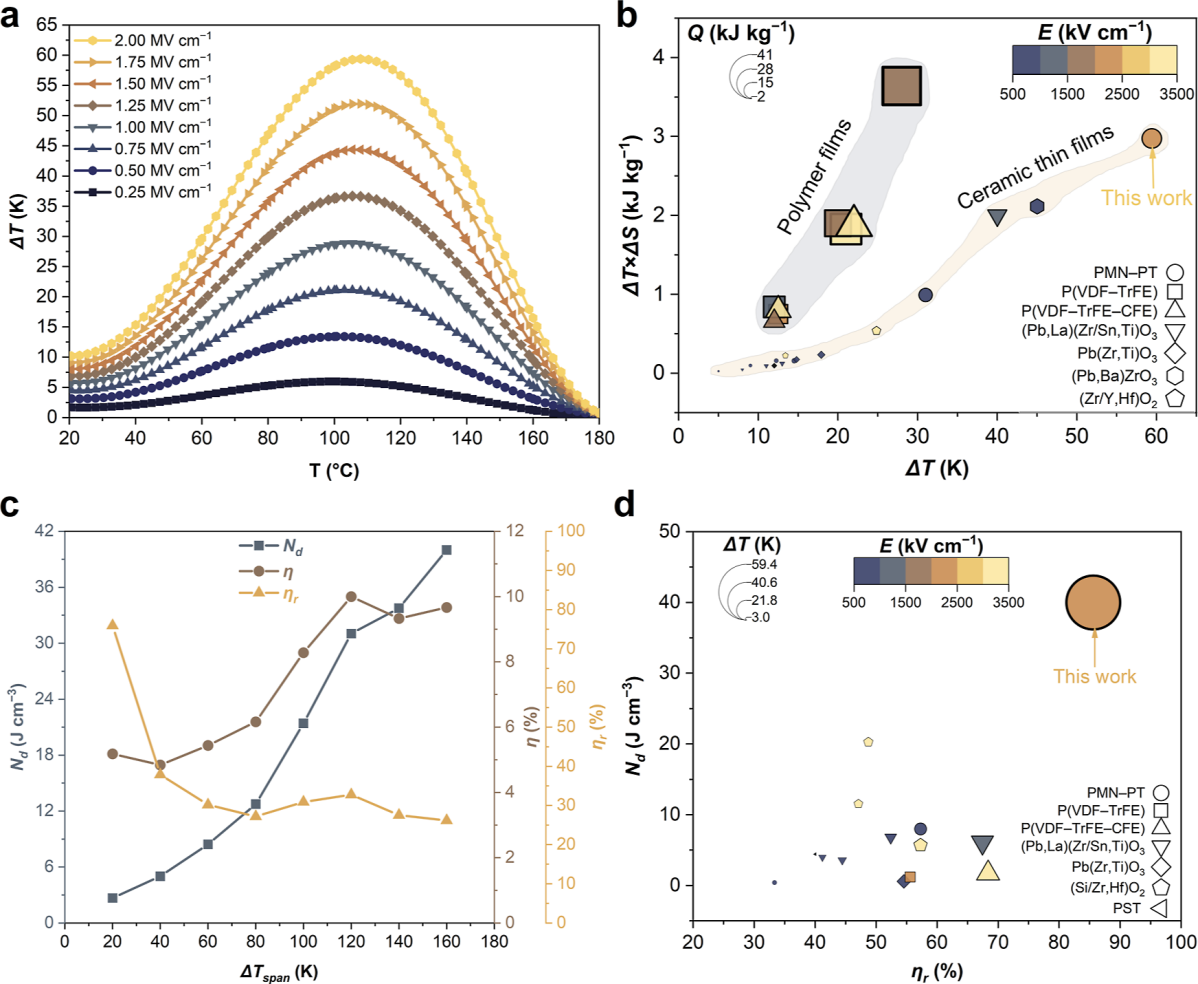
Electrocaloric effect and pyroelectric energy harvesting of Sm-PMN–30PT film. Thermal evolution of the (a) Δ*T* at different *E* and (b) comparison of Δ*T*, Δ*T* × Δ*S*, and *Q* achieved under different *E* in the current study with other conventional electrocaloric caloric materials reported works (the corresponding data are shown in Table S1). The symbol size and fill color refer to the values of *Q* and *E*, respectively. (c) Evolution of *N*_d_, η, and η_r_ at various Δ*T*_span_ between 0 and 160 °C at 2 MV cm^–1^. (d) Estimated energy harvesting ability (*N*_d_ and η_r_) for several materials showing outstanding electrocaloric ability (the corresponding data are shown in Table S2). The symbol size and the fill color refer to the values of Δ*T* and *E*, respectively. When not directly available, *Q*_ECE_ is calculated using *Q*_ECE_ = ρ*C*_p_Δ*T*.

The capability of the Sm-PMN–30PT film to interconvert electrical and heat energy via two fundamental mechanisms, i.e., electrocaloric and pyroelectric effects, was investigated (calculation details can be found in the Supporting Information). Due to the low thermal mass of thin films with respect to the substrate, an indirect approach involving of the Maxwell relation is generally employed to assess the ECE in thin films.^3,24,26^ The thermal evolution of the adiabatic temperature change (Δ*T*) under different applied electric fields is shown in Figure 3a. By increasing the electric field, Δ*T* is augmented to reach giant values of 59.4 K (∼1.18 K V^–1^), corresponding to isothermal entropy change (Δ*S*) of 50.1 J kg^–1^ K^–1^, around Curie temperature (*T*_C_ = 107 °C) at 2 MV cm^–1^. These values surpass those reported by Mischenko et al. in PbZr_0.95_Ti_0.05_O_3_ films (Δ*T* = 12 K and Δ*S* = 8 J kg^–1^ K^–1^).^25^ To emphasize that both Δ*T* and Δ*S* are important in cooling systems, Δ*T* × Δ*S* is used to compare the performance of the electrocaloric materials. The obtained Δ*T* × Δ*S* value of the Sm-PMN–30PT is ∼2.98 kJ kg^–1^ is three times higher compared to the state-of-the-art undoped PMN–PT films (∼0.99 kJ kg^–1^),^26^ and enhanced than other ceramic thin films reported in the literature (Table S1).^27−32^ Moreover, the suitability of the electrocaloric material for the application in solid-state cooling technologies is typically evaluated by the refrigerant capacity (RC),  and the coefficient of performance ,^33,34^ where *T*_L_ and *T*_H_ are the cold and hot temperatures accessed via the EC effect, and *Q* is the isothermal heat. At the maximum electric field, these parameters are estimated to be RC = 4.8 kJ kg^–1^ (i.e., 38.4 J cm^–3^) by taking *T*_L_ = 0 °C and *T*_H_ = 160 °C and COP = 4.18 (with *Q* = 19 kJ kg^–1^). These values are higher than other ceramic thin films reported in the literature (Figure 3b).^25,30,34−40^ Additionally, thermally stable ECE is observed in Sm-PMN–30PT RFE thin films due to enhanced relaxor behavior. For example, while the PBZ film shows a Δ*T* of 45 K at 17 °C with an 80% drop after 10 °C from the *T*_C_, the Sm-PMN–PT thin film, with a colossal ECE of 59.4 K, experiences less than a 1% drop in Δ*T* after 10 °C from the *T*_C_, owing to its enhanced relaxor behavior. In conclusion, these findings indicate that the Sm-PMN–30PT RFE thin film, with giant ECE, shows tremendous potential in utilization as electrical refrigeration and solid-state cooling technologies.

We evaluated the ability of Sm-PMN–30PT film to harvest waste heat to generate clean electrical energy via the pyroelectric effect. The amount of harvested energy density (*N*_d_) between 0 and 160 °C, corresponding to Δ*T*_span_ of 160 °C, by applying 2 MV cm^–1^ is 40 J cm^–3^, which is colossal compared to other pyroelectric harvesters reported in the literature (Table S2).^5,41−44^ Besides, we assessed how efficiently our film converts thermal heat into electric energy. The energy density *N*_d_, efficiency η and the scaled efficiency η_r_, respectively, of the Olsen cycle as a function of the temperature span of Sm-PMN–30PT are shown in Figure 3c. The efficiency η reaches 10.0%, corresponding to 32.7% of η_r_ at Δ*T*_span_ = 120 K. However, η_r_ reaches values as large as 75.9% for a Δ*T*_span_ of 20 K, between 0 and 20 °C, at an electric field of 2 MV cm^–1^. It is worth recognizing the positive role of the phase transition on efficiency. Indeed, the best values of η and η_r_ are nearly all obtained around the Curie temperature (*T*_C_), due to enabling the occurrence of the field-induced phase transition.^45^ For comparison to the literature, we have calculated the above values at *E* = 300 kV cm^–1^ between 0 and 20 °C (Δ*T*_span_ = 20 K) and found η_r_ = 15.2%. This value is about 2-fold larger than the state-of-the art PMN–32PT thin films (η_r_ = 8%) at the same *E* and Δ*T*_span_.^5^ Accordingly, the Sm-PMN–30PT could be applicable for active heat extraction from an electronic junction since the phase transition is close to 100 °C.^6^

If the electrocaloric energy is much larger than the energy stored in the sample’s heat capacity during the heating sequence, then the final conversion ratio exactly equals Carnot’s. The consequence is that, for a very small temperature difference, it is possible to harvest energy with Carnot’s efficiency (in contrast to thermoelectric conversion).^46^ Since Sm-PMN–30PT exhibits a huge electrocaloric effect, a large pyroelectric energy efficiency is expected, thus we can rewrite .^6,46^ To compare our findings to others reported in the literature, we consider Δ*T*_span_ = 10 K, hence, η_r_ is estimated to be 85.5%, which is far exceeding the state-of-the-art PbZr_0.95_Ti_0.05_O_3_ thin film (η_r_ ≈ 56%)^25^ and PVDF-TrFE-CFE thin polymer films (η_r_ ≈ 60–70%), as shown in Figure 3d.^6,29,33,42,47−49^

As discussed previously, the dielectric and thermal breakdown strengths are the bottleneck in ceramic oxide thin films. There is a growing body of literature that recognizes the importance of the BDS in the capacitive energy storage,^50^ however the performance of the thin film is limited by the thermal breakdown strength. This latter significantly decreases the magnitude of the electrocaloric and pyroelectric effects. Accordingly, we focused on the applied electric field to compare these two effects in Sm-PMN–PT thin film to literature (Figure 3b–d). As can be seen, in most oxide thin films, excluding HfO_2_, the applied electric field is around 1, and ∼0.9 MV cm^–1^ in the case of PMN–PT films,^31^ which resulted in moderate Δ*T* and *N*_d_ values. Thus, the Sm-doping improved also the thermal breakdown strength of PMN–PT.

### Phases and Nanodomain Structures

**Figure 4:**
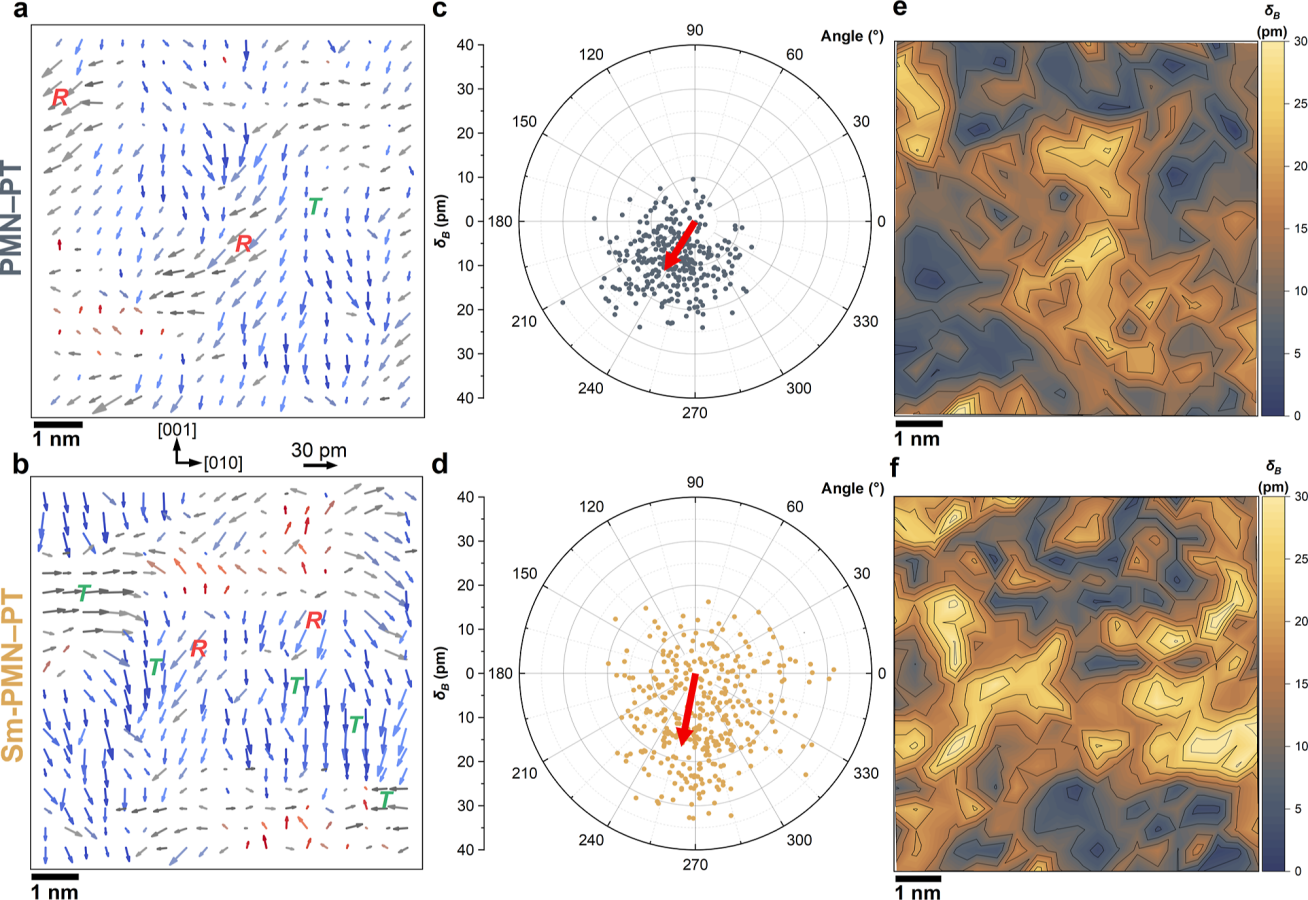
Phases and nanodomain structures of PMN–30PT and Sm-PMN–30PT films. B-site atom displacements from the center of four neighboring A-site atoms of the perovskite unit cell (see schematics on the top-left side) for (a) PMN–30PT and (b) Sm-PMN–30PT films, respectively. The length of the arrows indicates the magnitude of the relative displacement of B-site atoms from the ideal position. Polar plots with arrows indicate the average magnitude and angle of the B-site atom displacements for (c) PMN–30PT and (d) Sm-PMN–30PT films, respectively. B-site atom displacements’ magnitude maps (with contour lines) for (e) PMN–30PT and (f) Sm-PMN–30PT thin films, respectively.

To uncover the reason for the enhancement of the piezoelectric, ferroelectric, electrocaloric and pyroelectric properties upon Sm-doping, we investigated the intimate relationship between the average symmetry, local heterogeneity, and domain structure in PMN–30PT and Sm-PMN–30PT films using high-angle annular dark field imaging with scanning transmission electron microscopy (HAADF–STEM; original HAADF images are shown in Figure S10). We analyzed the B-site atom off-center displacements (represented by arrows) within their A-sites cages (Figures 4 and S11a), as well as the A-sublattice distortions arising from the spatial disorder of the A-site atoms (Figures S10b, S12 and S13). Both samples exhibited nonisolated nanodomain structures with diameters between approximately 2 and 5 nm (groups of atoms with correlated displacement orientation). Specifically, PMN–30PT film demonstrated small and relatively uniform B-site atom displacements (δ_B_) with the presence of *R* distortions and scarce *T* distortions, with an average δ_B_ of 12.3 ± 5.7 pm along [01̅1̅] direction (Figure 4a–c). In contrast, the Sm-PMN–30PT sample displayed the coexistence of *R* and *T* distortions, with larger δ_B_ (15.2 ± 7.1 pm) and slightly tilted toward the [001̅] direction corresponding to the tetragonal system (Figure 4b–d). The B-site atom displacements’ magnitude maps of PMN–30PT and Sm-PMN–30PT films are shown in Figure 4e,f. Notably, in the Sm-PMN–30PT sample, the *R* and *T* clusters interact directly with each other, forming a unique slush-like polar structure with a multidomain state.^15,51^ This configuration is typical of PMN–PT materials, where a multidomain state with various low-angle domain walls separates small polar domains, without a nonpolar matrix.^15^ In this structure, the displacement vectors in adjacent unit cells gradually rotate between the *R* and *T* nanoregions (Figure S12). Such a bridging domain mechanism can significantly reduce the domain wall energy, thus inducing the observed domain miniaturization with high-density and low-angle domain walls.^16^ It is worth mentioning that regions showing small δ_B_ in the image plane can possess significant δ_*B*_ in the direction perpendicular to the image plane.

**Figure 5:**
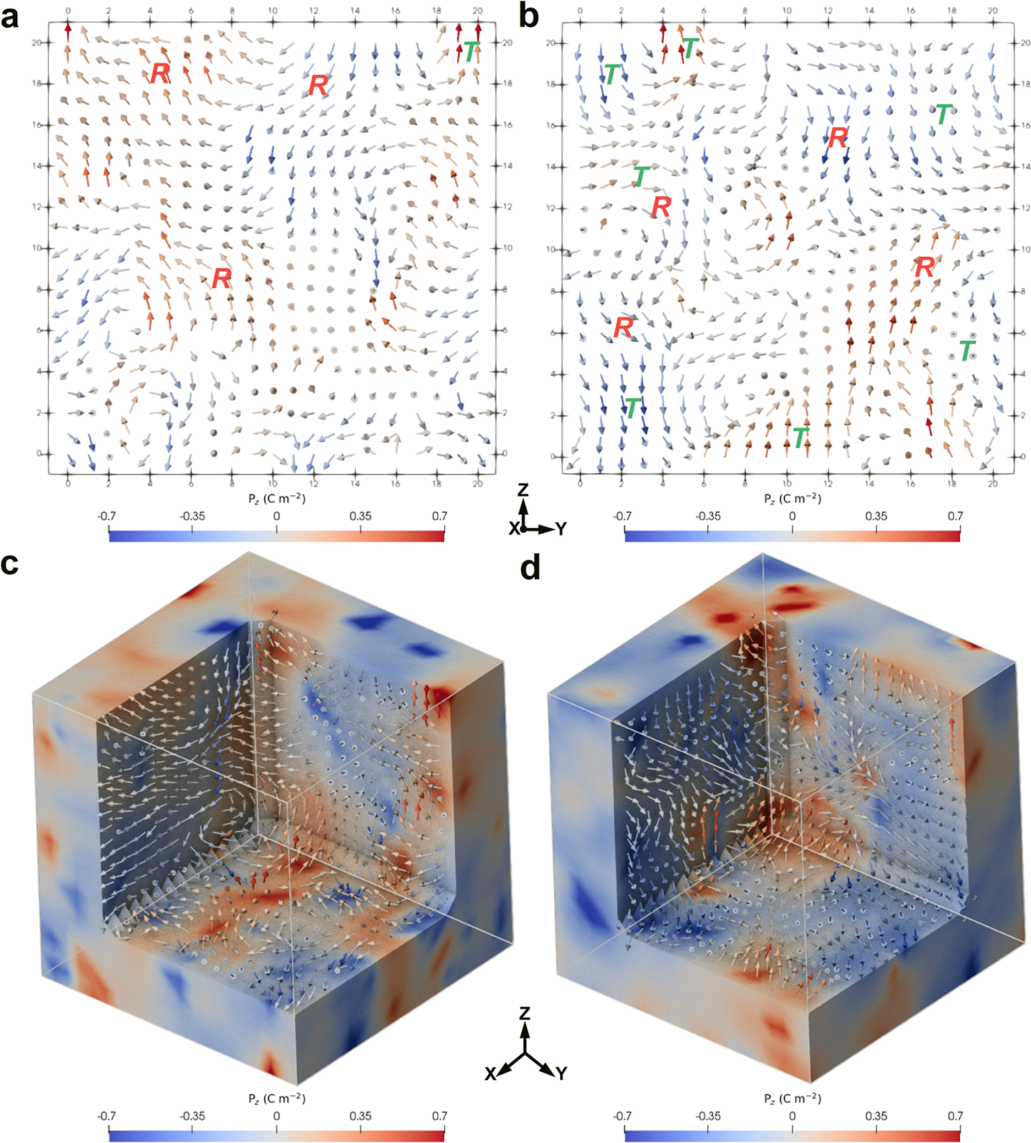
Phase-field modeling of the nanodomain structures of PMN–30PT and Sm-PMN–30PT films. Modeling of the two-dimensional (a,b) and three-dimensional (c,d) slush-like nanodomain structure of PMN–30PT and Sm-PMN–30PT films, respectively.

An increase in local structural disorder resulting from Sm-doping led to the creation of a quenched random local field.^10^ We investigated the effect of the local electric field in PMN–30PT and Sm-PMN–30PT films using a phase field modeling with randomly localized point distribution of the fields coupled with polarization (Figure 5). The fields, aligned with the *x*, *y*, or *z* directions, tend to create *a*- or *c*-domains. Their local distribution impacts only a small region of 2–3 nm, forming a slush-like polar structure similar to those observed by STEM. Furthermore, we modeled the evolution of the domain structure in the electric field *E*, as shown in Movies S1 and S2. Under the application of the field, the domains in PMN–30PT and Sm-PMN–30PT films align with the field. When the field is reset to zero, the PNDs easily revert to their random orientations due to their small sizes, resulting in slim polarization hysteresis loops with low *P*_r_ values.^52^ The experimental and phase-field modeled *P*–*E* hysteresis loops at 1 MV cm^–1^ of PMN–30PT and Sm-PMN–30PT films are shown in Figure S16.

## Conclusion

In summary, Sm-doping creates higher local structural heterogeneity and enhances the MPB characteristics, where several phenomena exist, including the instability of the polarization vector against rotation, a divergence in transverse susceptibility associated with extrema in electromechanical properties, a reduction of domain size, presence of low-angle domain walls, conditions favoring the stabilization of phases with lower symmetry, and the metastable coexistence of multiple phases.^53^ The coexistence of different ferroelectric structures with varying symmetries provides considerable scope for polarization rotation via a reduction of polarization anisotropy, significantly impacting the domain structure. Furthermore, the extremely high density and the low-angle domain walls give rise to greater flexibility for polarization rotations over a wide temperature range, which proves the robust thermal stability of the energy density in the Sm-PMN–30PT thin film.^15^ Additionally, the coexistence of *R*, and *T* phases makes the lattice more frustrated and leads to significant Δ*S* and Δ*T* values.^54^ Furthermore, as the domain wall density increases, the macroscopic average electrocaloric response can be enhanced multiplicatively.^55^ Besides, the ability of the Sm-PMN–30PT thin film to withstand high electric fields at high temperatures was crucial in boosting thermal energy harvesting and solid-state cooling performances. As a result of such phase degeneracy, the energy storage, piezoelectric, and electrocaloric properties of the Sm-PMN–30PT thin film are enhanced.

The design of high-density polymorphic nanodomains (slush-like polar structure) and low-angle domain walls has significantly boosted the capacitive energy storage, electrocaloric effect and pyroelectric energy density. This strategy holds wide-ranging potential for other RFEs by enabling the nanodomain structures’ manipulation to develop robust multifunctional materials.

## Supporting Information Available

Experimental procedures; structural, chemical compositions, piezoelectric, dielectric and ferroelectric properties of the thin films; dielectric breakdown strength; energy storage stability against fatigue and frequency; details about the electrocaloric and pyroelectric effects’ calculation; atomic resolution HAADF-STEM images; A-site positional disorder and local degree of tetragonality; tables comparing the electrocaloric effect and the pyroelectric energy harvesting performances; two movies showcasing the evolution of the domain structure in the electric field (PDF)
(MP4)
(MP4)
