# Supplementary material for: Thermally Stable Capacitive Energy-Density and Colossal Electrocaloric and Pyroelectric Effects of Sm-Doped Pb(Mg1/3Nb2/3)O3–PbTiO3 Thin Films
Source: J Am Chem Soc. 2024 Nov 18;146(47):32595–604. doi: 10.1021/jacs.4c11555 (PMC11613449; doi:10.1021/jacs.4c11555)
Supplement: Supplementary file 1 — ja4c11555_si_001.pdf [file ja4c11555_si_001.pdf]

Supporting Information

Thermally-stable capacitive energy-density and colossal electrocaloric and pyroelectric effects of Sm-doped $Pb(Mg_{1/3}Nb_{2/3})O_3–PbTiO_3$ thin films

Zouhair Hanani[1,*], Jamal Belhadi[2], Urška Trstenjak[1], Nick A. Shepelin[3], Vid Bobnar[4], Hana Uršič[5], Nina Daneu[1], Nikola Novak[4], David Fabijan[1], Anna Razumnaya[4], Yuri Tikhonov[2], Thomas Lippert[3,6], Zdravko Kutnjak[4], Gertjan Koster[1,7], Igor Lukyanchuk[2], and Matjaž Spreitzer[1,*]

[1]Advanced Materials Department, Jožef Stefan Institute, Jamova cesta 39, 1000, Ljubljana, Slovenia.
[2]Laboratory of Physics of Condensed Mater, University of Picardie Jules Verne, 33 rue Saint-Leu, Amiens 80039, France.
[3]Center for Neutron and Muon Sciences, Paul Scherrer Institute, Forschungsstrasse 111, 5232 Villigen PSI, Switzerland.
[4]Department of Condensed Matter Physics, Jožef Stefan Institute, Jamova cesta 39, 1000 Ljubljana, Slovenia.
[5]Electronic Ceramics Department, Jožef Stefan Institute, Jamova cesta 39, 1000, Ljubljana, Slovenia.
[6]Department of Chemistry and Applied Biosciences, ETH Zürich, 8093 Zürich, Switzerland.
[7]MESA+ Institute for Nanotechnology, University of Twente, 7500 AE Enschede, The Netherlands.

*Corresponding authors: email: zouhair.hanani@ijs.si (Z.H.); matjaz.spreitzer@ijs.si (M.S.)

1. Experimental procedures

1.1. Growth of the thin films using pulsed-laser deposition

Pure and 2 at. % Sm-doped PMN–30PT targets, abbreviated as PMN–30PT and Sm-PMN–30PT, respectively, were prepared in-house using the columbite route. 20 mol% PbO and 10 mol% MgO excess were used to ensure the stoichiometry of the films. (100)-$SrTiO_3$ single-crystal substrates (STO, CrysTech GMBH) were surface treated, at 1000°C for 1h under O_2-atmosphere, to obtain an atomically-flat surface. Both thin-film heterostructures were grown using pulsed-laser deposition (PLD) in an on-axis geometry with a target-to-substrate distance of 55 mm, using a KrF excimer laser (248 nm, LPX 300, Coherent). Before growing PMN–30PT and Sm-PMN–30PT thin films, a 35 nm-thick layer of $LaNiO_3$ (LNO, Solmates) serving as bottom electrode, was deposited on STO substrates. LNO layer was deposited at 570°C in a dynamic oxygen pressure of 0.13 mbar with a laser fluence of 2.5 J cm^{-2} and a laser repetition rate of 4 Hz, while the 250 nm-thick PMN–30PT and Sm-PMN–30PT thin films were grown at the same temperature under an oxygen partial pressure of 0.27 mbar, laser fluence of 2.25 J cm^{-2} and laser pulse frequency of 8 Hz. Following the growth, the samples were cooled down to room temperature at 10°C min^{-1} in a static O_2-pressure of 700 mbar.

1.2. Structural characterizations

The X-ray θ–2θ patterns and reciprocal-space maps (RSMs) were collected with an X-ray diffractometer (Empyrean, Malvern PANalytical) with CuKα1 radiation (λ = 1.5406 Å). A double-bounce Ge (220) hybrid monochromator was used on the incident-beam side. The diffracted beam in θ–2θ scans and RSMs was captured and analyzed by a PIXcel3D detector operating in 1D mode. The surface structure of the films was examined by an *in-situ* reflection high-energy electron diffraction (RHEED) system (STAIB Instruments) with an accelerating voltage of 30 kV, and the patterns were analyzed using kSA 400 software (k-Space Associates). In addition, the surface morphology of the samples was examined by atomic force microscopy (AFM, Veeco Dimension 3100 SPM) using silicon probes (OTESPA-R3, Bruker), and the topographical data was thoroughly analyzed with the help of Gwyddion AFM analysis software (gwyddion.net).[1]

1.3. HAADF-STEM experiments

Cross sectional scanning transmission electron microscopy (STEM) samples of ~45 nm in thickness were prepared using Focused Ion Beam (FIB, Helios Nanolab 650) with Ga^+ ions. The STEM experiments were performed using a 200 kV probe-aberration corrected atomic-resolution scanning transmission electron microscope (JEOL ARM200 CF, Jeol Ltd.) equipped with an energy-dispersive X-ray spectrometer (EDS, Jeol Centurio 100) for the analyses of chemical composition. The atomic-

resolved High-angle annular dark-field (HAADF) images were collected with a convergence angle of 24 mrad and a collection angle of 68 – 180 mrad. To minimize the influence of the specimen drift and scanning irregularities on the atomic column positions HAADF images were taken as a stack of 10 images; each frame was taken with pixel time of 1.6 μs (512 × 512 px) using DigiScan Stack Acquisition Tool in DigitalMicrograph. After the acquisition all images in the stack were aligned using cross-correlation and averaged to obtain low-noise, good quality STEM images using Stack Alignment in DigitalMicrograph. The central position associated with each atomic column was localized on the averaged HAADF images, taken in $[100]_{pc}$ zone axis (pc for pseudo cubic), using a two-dimensional Gaussian fitting procedure, and displacements for the B- and A-site columns were determined by measuring their displacements from ideal cubic positions. Image analysis and quantification were performed using atom column indexing[2] and custom Python scripts. Besides, the size of the polar-nanodomains (PNDs) were numerically found by Bayesian methods.[3]

1.4. PFM measurements

The local piezoelectric response of PMN–30PT and Sm-PMN–30PT thin films was studied by piezoresponse force microscopy (PFM) using an Asylum Research, Molecular Force Probe 3D AFM in dual AC resonance-tracking (DART) mode. A Pt-coated Si tip with a radius of curvature of ∼10 nm (OMCL-AC240TM-R3, Olympus, Japan) was employed. The spring constant and the resonance frequency of the cantilevers were 2 N m^{-1} and 70 kHz. Two scanning AC electric voltages of 6 and 10 V, and a frequency of 350 kHz were applied between the conductive AFM tip and LNO bottom electrode.[4]

1.5. Electrical measurements

The samples were measured in parallel plate capacitor geometry using an LNO-bottom electrode and sputtered gold, using shadow masks with holes of 100 and 500 μm in diameter, as the top electrode. The 100 μm-electrodes were used for dielectric and ferroelectric measurements, while the 500 μm ones were used to determine the piezoelectric properties of the films. The dielectric measurements were conducted from 20 Hz to 1 MHz and an AC amplitude of 100 mV using an LCR meter (4284, Agilent) with Cascade DCP-HTR probes. Polarization–electric field (P–E) hysteresis loops were recorded at a frequency of 100 Hz with a triangular excitation signal using a ferroelectric test system (TF Analyzer 2000E, aixACCT) equipped with a high-voltage amplifier. Fatigue measurements were performed at room temperature by completing bipolar P–E hysteresis loops at 100 Hz after applying a 10-kHz triangular waveform at 1 MV cm^{-1} up to one billion cycles. The bias-field dependent dielectric constant curves (calculated from the capacitance–voltage (C–V) characteristics) were measured at a small ac signal amplitude of 50 mV by applying a dc bias field of ±0.2 MV cm^{-1} using

the same ferroelectric test system. The temperature of the sample was controlled by a thermal controller (PE95/T95/LNP95, Linkam) with an accuracy of 0.1°C. The piezoelectric characteristics of the capacitors were determined using a double-beam laser interferometer (aixDBLI, aixACCT). The piezoelectric coefficient (d_{33}) was measured at different dc fields by superimposing a small ac signal with an amplitude of 100 mV and frequency of 1 kHz on a stepwise dc signal with maximal amplitude corresponding to ±0.6 MV cm^{-1}.

1.6. Phase-field modelling

Numerical simulations of slush domains and polar nanoregions in relaxor thin films are based on the minimization of the Ginzburg-Landau free energy functional [5] in which the elastic and electrostatic effects are taken into account:

$$F = \int \Big(\left[\alpha_i(T) P_i^2 + \alpha_{ij} P_i^2 P_j^2 + \alpha_{ijk} P_i^2 P_j^2 P_k^2 \right]_{i \le j \le k} + \frac{1}{2} G_{ijkl} (\partial_i P_j)(\partial_k P_l) - \frac{1}{2} \varepsilon_0 \varepsilon_b (\nabla \varphi)^2 + (\partial_i \varphi) P_i + P_i E_i + \frac{1}{2} C_{ijkl} u_{ij} u_{kl} - C_{ijkl} Q_{klmn} u_{ij} P_m P_n \Big) \, d^3 r \qquad (1)$$

Here we assume the tensor summation over the repetitive indices that takes the cartesian components *x*, *y*, *z* (or 1,2,3).

Functional[5] comprises the Ginzburg-Landau energy[6] given in the first square brackets. The second term is the polarization gradient energy.[7] The third and fourth terms represent the electrostatic energy.[8] The fifth term is a coupling between the polarization field and a random local field. The last two terms correspond to the elastic energy. The electrostatic potential and strain tensor are denoted as φ and u_{ij} respectively. The value of the vacuum permittivity ε_0 is 8.85×10^{-12} CV^{-1}m^{-1} and the value of the background dielectric constant ε_b is 10.[9] The numerical values of the Ginzburg-Landau expansion coefficients α_{ijk}, gradient energy coefficients G_{ijkl}, elastic stiffness tensor C_{ijkl} and tensor of electrostrictive coefficients Q_{ijkl} are given below.

The distribution of the electrostatic potential φ and the elastic strains u_{ij} is found from the respective electrostatic (with screening) and elastic equations:

$$\varepsilon_0 \varepsilon_b \nabla^2 \varphi = \partial_i P_j \qquad (2)$$

$$C_{ijkl} \partial_i (u_{kl} - Q_{klmn} P_m P_n) = 0 \qquad (3)$$

The parameters of the functional[5] for PMN–30PT at room temperature were taken as: α_1 =0.745(T − 385.7)×10^5 C^{-2}m^{2}N, α_{11} = 0.264×10^8 C^{-4}m^{6}N, α_{12}= −0.295×10^8 C^{-4}m^{6}N, α_{111} = 0.5567×10^9 C^{-6}m^{10}N, α_{112} = 1.333×10^9 C^{-6}m^{10}N, α_{123} = 0.24×10^9 C^{-6}m^{10}N; Q_{1111} = 0.055 C^{-2}m^4, Q_{1122} = −0.023 C^{-2}m^4, and Q_{1212} = 0.03 C^{-2}m^4; C_{1111} = 0.9×10^{11} m^{-2}N, C_{1122} = 0.545×10^{11} m^{-2}N, and C_{1212} = 0.5×10^{11}

$m^{-2}N$; G_{1111} = 0.213×10^{-11} $C^{-2}m^4N$, G_{1122} = 0, and G_{1212} = 0.106×10^{-11} $C^{-2}m^4N$. All of them were collected from;[10] components of the elastic stiffness being calculated from compliance tensor and the standard procedure of recalculation of second-order coefficients α_{ij} for the strain-free case being applied.[11]

The parameter set of Sm-doped PMN–30PT differs from that for PMN–30PT by the coefficients α_{111}=0.2783×10^9 $C^{-6}m^{10}N$, α_{112}=0.665×10^9 $C^{-6}m^{10}N$ and α_{123} = 0.12×10^9 $C^{-6}m^{10}N$ that we take to fit the polarization curve $P(E)$ in the high-field region where all the system is polarized.

The non-linear differential relaxation equation is used to find the minima of the free-energy [5]:

$$-\gamma\frac{\partial P}{\partial t}=\frac{\delta F}{\delta P} \qquad (4)$$

Here γ is a time-scale parameter which is taken to be equal be equal unity for convenience. The non-linear part of the equations is accompanied by two linear systems of equations defined by the screened Poisson equation (2) and the equation of linear elasticity (3).

The phase-field simulations were conducted with the help of the FEniCS software package.[12] Two- and three-dimensional rectangular computational regions are represented by structured triangular and tetrahedral finite element meshes, respectively, that were created with the 3*D* mesh generator *gmsh*.[13] The solutions for **P**, φ and u_{ij} was sought in the functional space of piecewise linear polynomials.

The approximation of the time derivative on the left-hand side of equation (4) is accomplished by BDF2 variable time stepper.[14] The initial condition for polarization at the first-time step is a random distribution of the polarization vector components in the range of −10^{-6} to 10^{-6} Cm^{-2}. Newton method with line search is used to solve the non-linear system arising from equation (4). To solve the linear system on each non-linear iteration and systems defined by equations (2) and (3), the generalized minimal residual method with restart is used (petsc-user-ref,petsc-web-page).

Thin film structure is represented by an unstructured tetrahedral cube with the side of 20 nm, where variables **P** and φ are constrained with periodic boundary conditions in the x and y directions. Simulation of the P–E hysteresis loop was accomplished by assuming the Dirichlet boundary conditions on the φ at the top and bottom sides of the cube. Built-in electrostatic field was introduced as a pointwise vector field with random distribution that occupies a specific amount of the film's volume. At each point the direction of the electrostatic field vector is randomly chosen from six possible tetragonal directions and eight directions corresponding to *R*-phase. The average magnitude of the in-site fields is taken as 5.8×10^2 kV mm^{-1}, which approximately corresponds to the electric

fields, $E_i = \frac{q}{4\pi\varepsilon_0\varepsilon_{eff}}$, produced by the uncompensated charges $q \approx \pm1.6\times10^{-19}$ C of the nearest ions Mg^{2+} and Nb^{5+} located at the distance of the unit lattice parameter $a \approx 0.4$ nm.

2. Structural properties and chemical compositions of the thin films

After growth, structural studies of PMN–30PT and Sm-PMN–30PT thin films were completed by X-ray diffraction. High-quality, (00l)-oriented, and fully epitaxial solid solution films were obtained (Figure S1a). To further investigate the structural properties of the grown films, i.e., in-plane cell parameters, strain state, and domain structures, a reciprocal space map of the asymmetric $(0\bar{1}3)_{pc}$ reflection was recorded and shown in Figure S1, b and c. It was found that the LNO bottom electrode is fully strained (same Q_x as the substrate), while the PMN–30PT and Sm-PMN–30PT layers are relaxed with respect to the STO substrate. The lattice parameters calculated from RSM of PMN–30PT and Sm-PMN–30PT thin films are (a = 4.0259 Å and c = 4.0274 Å) and (a=4.0215 Å and c=4.0305 Å), respectively. Accordingly, the Sm-PMN–30PT has high degree of tetragonality (c/a) compared to the pure PMN–30PT sample, because Sm^{3+} favors the tetragonal phase and shifts the composition toward the morphotropic phase boundary (MPB).[15] The surface structure of PMN–30PT and Sm-PMN–30PT thin films was monitored by *in situ* reflection high energy electron diffraction (RHEED), and both films showed the epitaxial growth of the films in 2D and in combination with a 3D combination as the streaks overlapped with faint 3D transmission spots (Figure S1, d and e). Besides, the surface topography of both films, examined by atomic force microscopy (AFM) over a scan area of 2 μm × 2 μm, revealed a rough surface in the PMN–30PT with a root-mean-square roughness (RMS) of 1.45 nm and smoother one in the Sm-doped film (RMS = 0.85 nm) (Figure S1, f and g). To study the microstructures of the Sm-PMN–30PT thin film in cross-section, STEM was used, and a low-magnification cross-sectional image of the sample is shown in Figure S1i. The LNO layer with a thickness of 35 nm is characterized by a high density of Ruddlesden-Popper defects that mainly extend perpendicular to the substrate. As a result, the LNO layer develops a columnar texture with a rough surface that provides many nucleation sites for the growth of the Sm-PMN–30PT layer. Single point elemental mapping using energy dispersive X-ray spectroscopy (EDS) spectra of PMN–30PT and Sm-PMN–30PT thin films are presented in Figure S2a, and elemental mapping using EDS reveals uniform detection of Sm, Pb, Mg, Nb and Ti elements across the film without evidence segregation (Figure S2c).

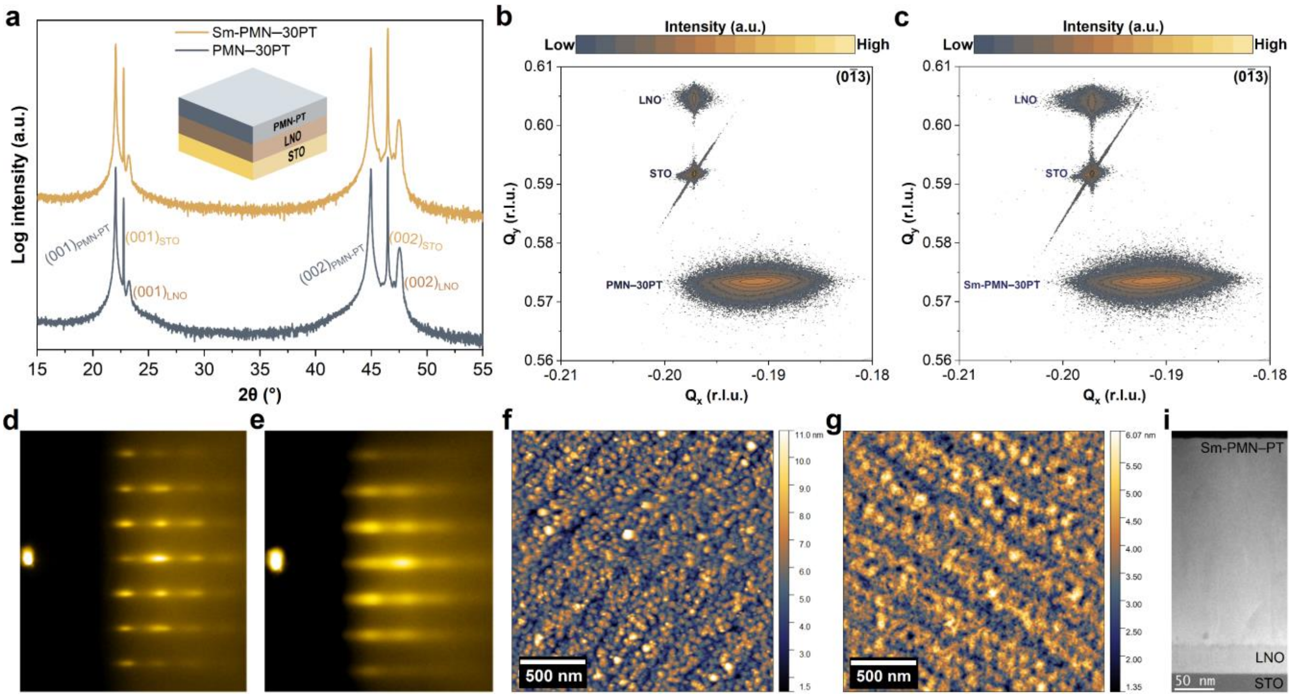

Figure S1. Effect of Sm-doping on the structural properties. (a) XRD patterns (inset shows a schematic illustration of the PMN–PT/LNO/STO heterostructure), (b, c) RSM around the $(0\bar{1}3)_{pc}$ reflection, (d, e) RHEED patterns, and (f, g) AFM topography images of PMN–30PT and Sm-PMN–30PT thin films, respectively. (i) Bright field STEM cross-section micrograph showing the corresponding layers in the epitaxial Sm-PMN–30PT thin film.

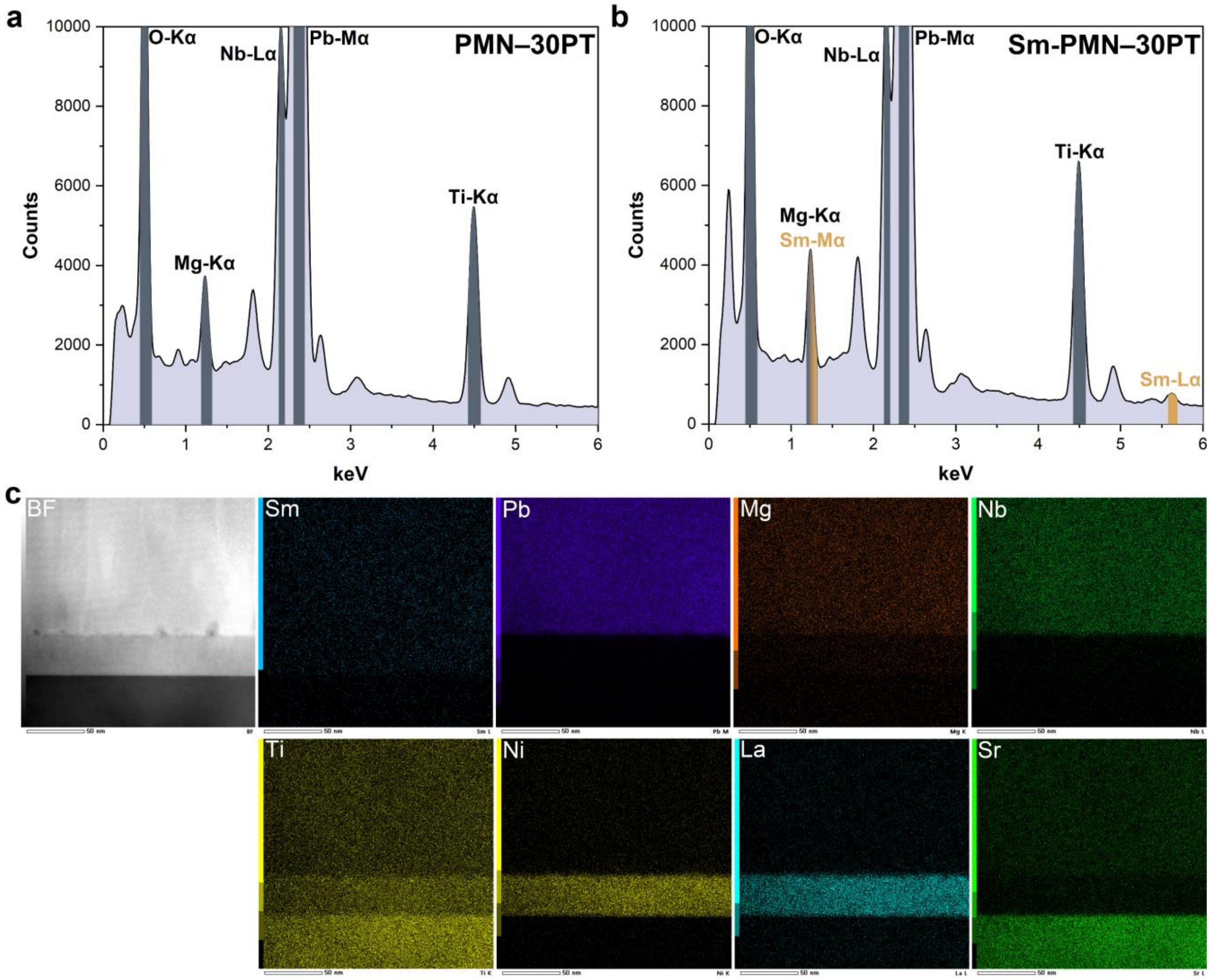

Figure S2. Chemical compositions of PMN–30PT and Sm-PMN–30PT thin films. Single EDS spectra of (a) PMN–30PT and (b) Sm-PMN–30PT thin films. (c) Bright-field TEM image and EDS elemental mappings indicate the uniformity of the Sm-PMN–30PT thin film.

3. Piezoelectricity of PMN–30PT and Sm-PMN–30PT thin films

To assess the effect of Sm-doping on the piezoelectric properties, the room-temperature d_{33}–E hysteresis of PMN–30PT and Sm-PMN–30PT films is shown in Figure S3. The Sm-doping resulted in improved d_{33} value of 70 pm V^{-1} compared to that of PMN–30PT (50 pm V^{-1}). Furthermore, the local piezoelectric response of both films was mapped by dual AC resonance tracking (DART) piezoresponse force microscopy (PFM), where the AFM topography (height and deflection) and PFM out-of-plane amplitude and phase images, which correspond to the magnitude and direction of the polarization vector in the film, respectively, are shown in Figure S4. In both samples, zero piezoresponse signal was observed at an AC drive amplitude of 6 V, indicating their relaxor-like behavior (Figure S4, a and b).[16] Nevertheless, when the samples were scanned at an AC drive amplitude of 10 V, no piezoelectric response was observed again in PMN–30PT, however started to appear in Sm-PMN–30PT (Figure S4, c and d). The fine-grain response noticed in Sm-PMN–30PT film can be attributed to clusters of piezo-active polar nano-regions that reorganize under an applied electric field as previously suggested for Pb-based relaxors.[16,17] In the PFM amplitude map, the bright contrast corresponds to areas with a larger piezoelectric response. These results indicate that the Sm-doping combined with the application of AC fields induce the relaxor-to-ferroelectric crossover behavior. It is worth mentioning that in Sm-PMN–30PT, the phase and amplitude overlap images (for example the area marked by the red dashed-rectangle in Figure S4d), which means that the brighter regions in the phase image represent domains with the normal component of polarization oriented upward, and the darker regions represent a downward orientation.[18]

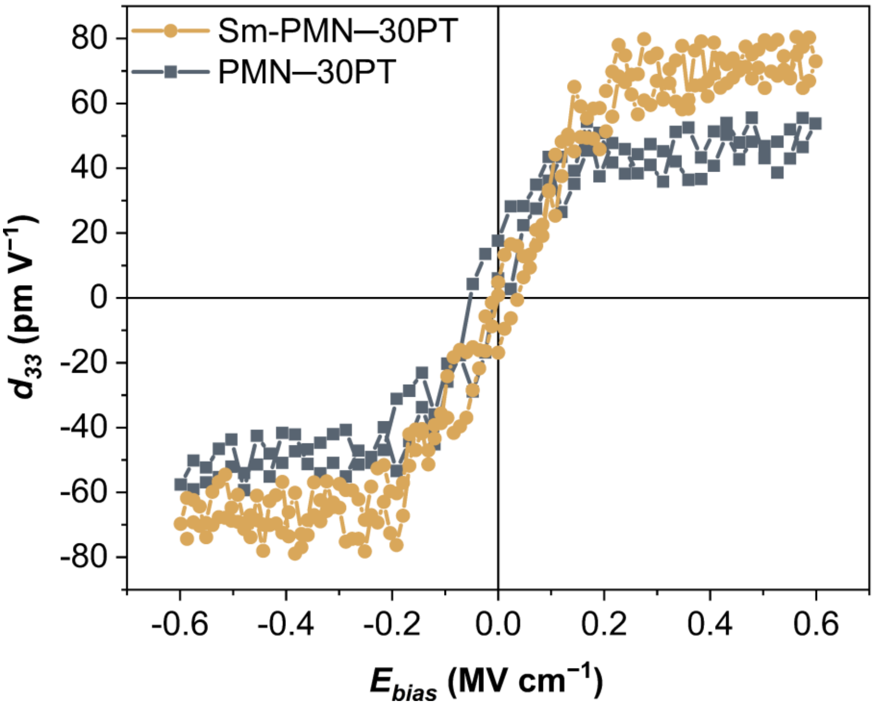

Figure S3. Effect of Sm-doping on the macroscopic piezoelectricity. Room-temperature d_{33}–E curves of Sm-PMN–30PT and PMN–30PT films.

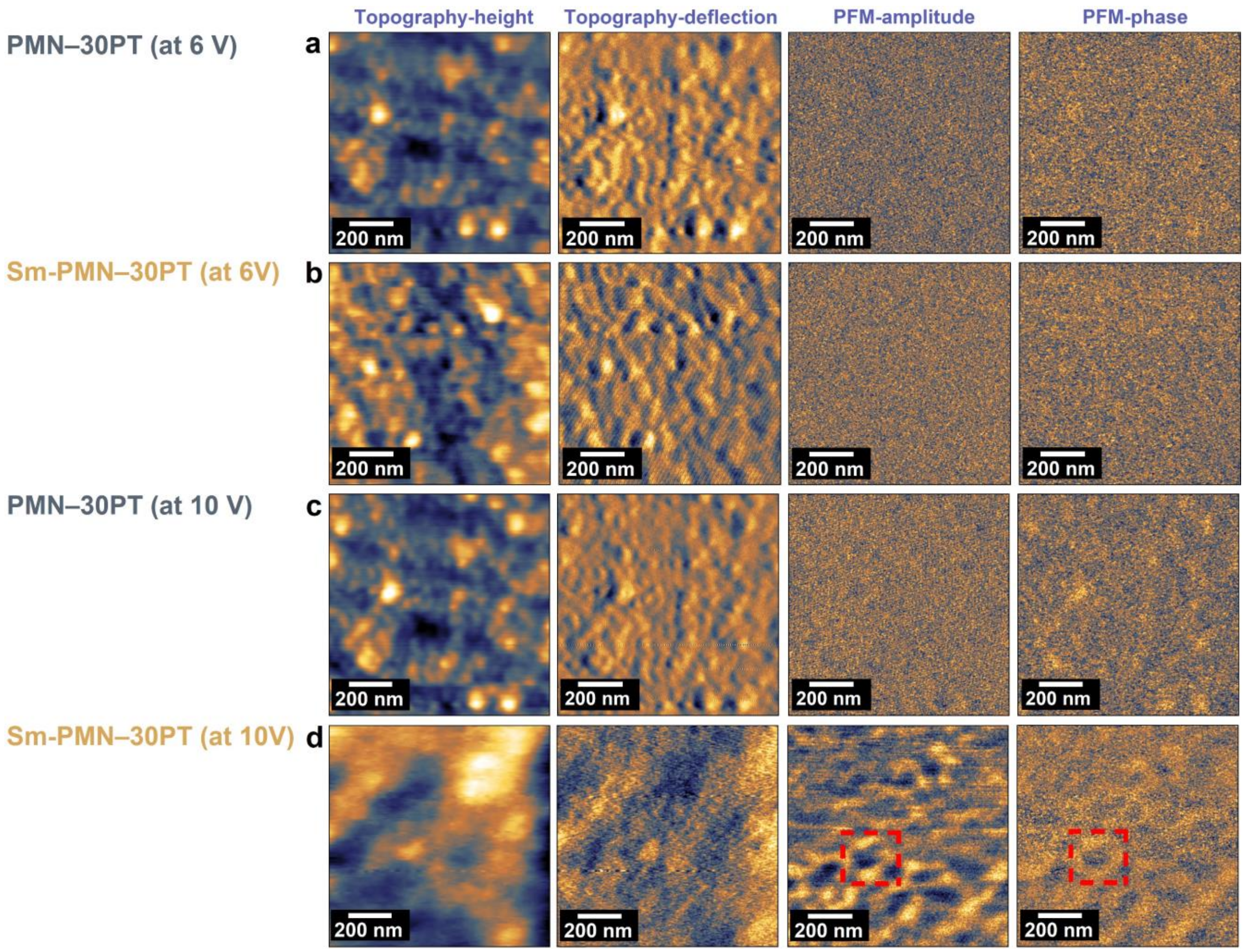

Figure S4. Effect of Sm-doping on the local piezoelectricity. AFM topography height and deflection, and PFM out-of-plane amplitude and phase images at an AC drive amplitude of 6 and 10 V for (a, c) PMN–30PT and (b, d) Sm-PMN–30PT thin films.

4. Dielectric and ferroelectric properties

The ferroelectricity of the films was assessed through *P–E* measurements at room temperature (Figure S5a). Both samples displayed typical characteristics of ergodic RFEs, as evidenced by non-saturated slim hysteresis loops, high maximum polarization (P_{max}) and low remnant polarization (P_r). Notably, PMN–30PT and Sm-PMN–30PT films showed negative and positive imprints of the *P–E* loop, respectively. At 1 MV cm^{-1}, the P_{max} and P_r values reached (50.5 μC cm^{-2} and 8.9 μC cm^{-2}) and (64.7 μC cm^{-2} and 3.5 μC cm^{-2}) in PMN–30PT and Sm-PMN–30PT films, respectively, indicating the enhanced relaxor behavior after Sm-doping. The frequency dependence of the dielectric permittivity (ε_r) and dielectric loss ($tan\ \delta$) of the pure and Sm-doped PMN–30PT thin films are shown in Figure S5b. A slight decrease of ε_r and the increase of $tan\ \delta$ at higher frequencies could arise in a relatively high resistance of the LNO bottom electrode.[19] Furthermore, ε_r versus bias-field of the pure and Sm-doped PMN–30PT thin films was recorded at 200 kV cm^{-1} (Figure S5c). In both samples, the ε_r–*E* curves showed a non-linear shape with double peaks around the coercive fields suggesting the ferroelectric behavior of our samples and can be explained in terms of increased domain walls' movement at the coercive fields. Furthermore, the noticeable asymmetry and the negative imprint of the ε_r–*E* response in the pure PMN–30PT thin film is due to an internal electric field oriented towards the bottom electrode in the undoped film. However, the ε_r–*E* curve in Sm-doped PMN–30PT thin film is slightly positively imprinted. Since LNO and Au electrodes were used in both samples, the origin of the internal electric field observed in the pure PMN–30PT thin film could be due to the strain gradient or non-homogenous spatial distribution of defects like oxygen vacancies.[20] Furthermore, the leakage current was measured at 1 MV cm^{-1}, and found to be reduced by about three orders of magnitude in Sm-PMN–30PT (Figure S5d), because the addition of the Sm^{3+} can reduce the conductivity of PMN–PT ceramics, indicating that the formation of $Sm_{\dot{P}b}$ defect annihilates and $V_{\ddot{O}}$ vacancy reduces the $V_{\ddot{O}}$ concentration.[21,22]

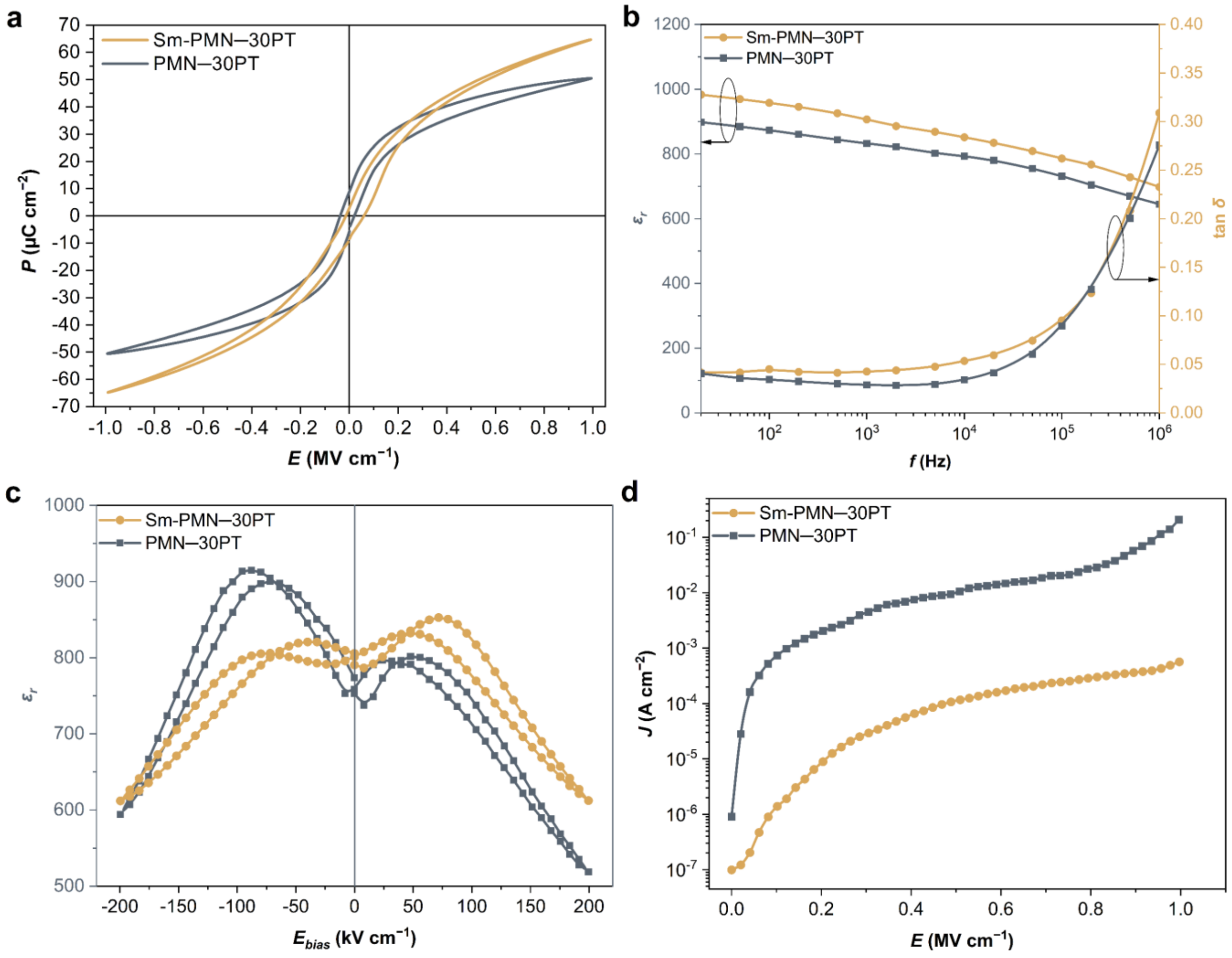

Figure S5. Effect of Sm-doping on the electrical properties. Room-temperature (a) *P*–*E* hysteresis loops at 1 MV cm^{-1}, (b) frequency dependent dielectric response, (c) dc-bias field dependence of the dielectric permittivity, (d) leakage current density of PMN–30PT and Sm-PMN–30PT thin films.

Figure S6 illustrates the temperature-dependence of ε_r and *tan δ* at various frequencies of the Sm-PMN–30PT thin film from –40 to 200°C. The sample exhibits only one dielectric anomaly associated with the ferroelectric–paraelectric (FE–PE) phase transition, together with a noticeable diffused ε_r – *T* response and frequency dispersion of the permittivity maxima.

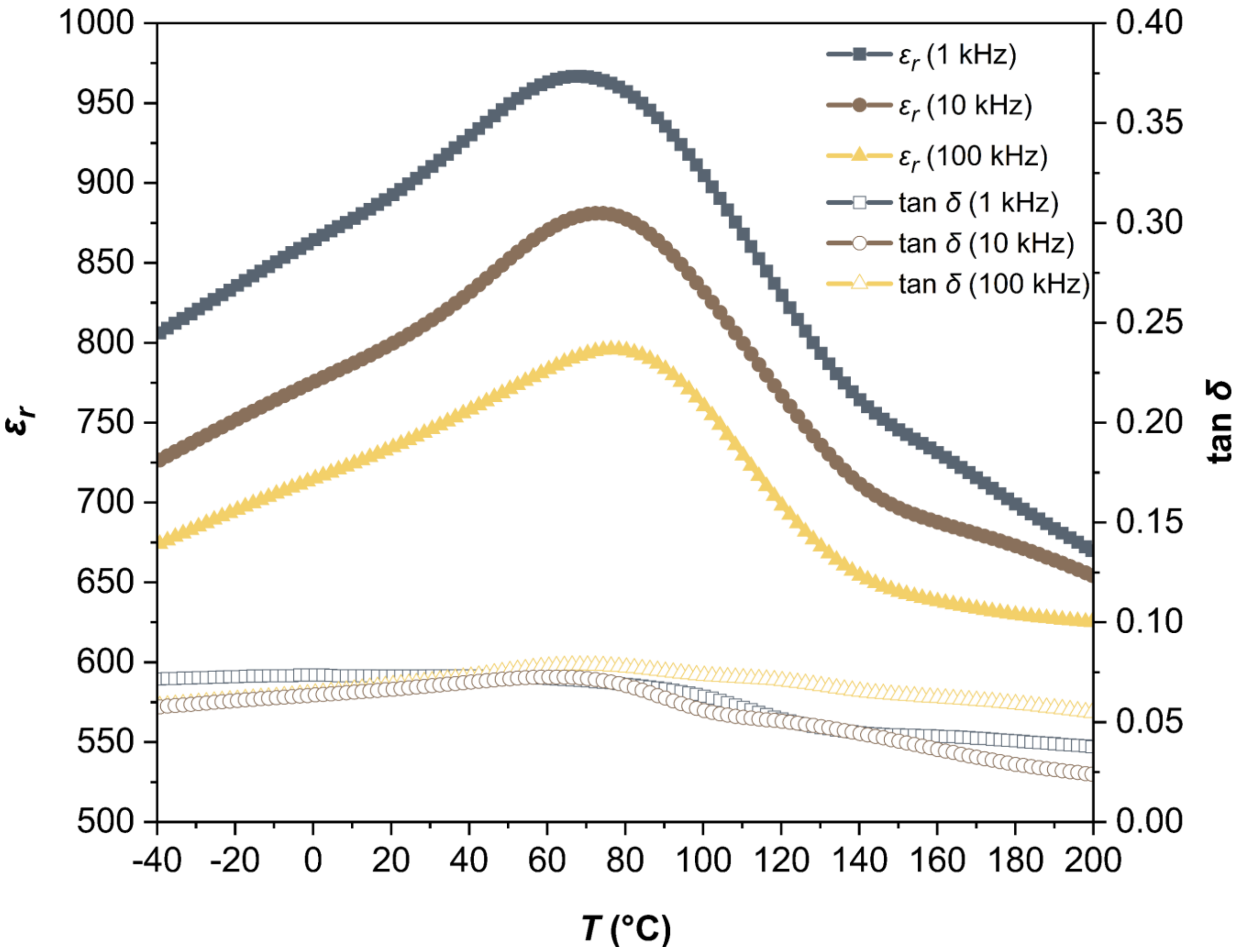

Figure S6. Temperature-dependence of dielectric properties at different frequencies of Sm-PMN–30PT thin film.

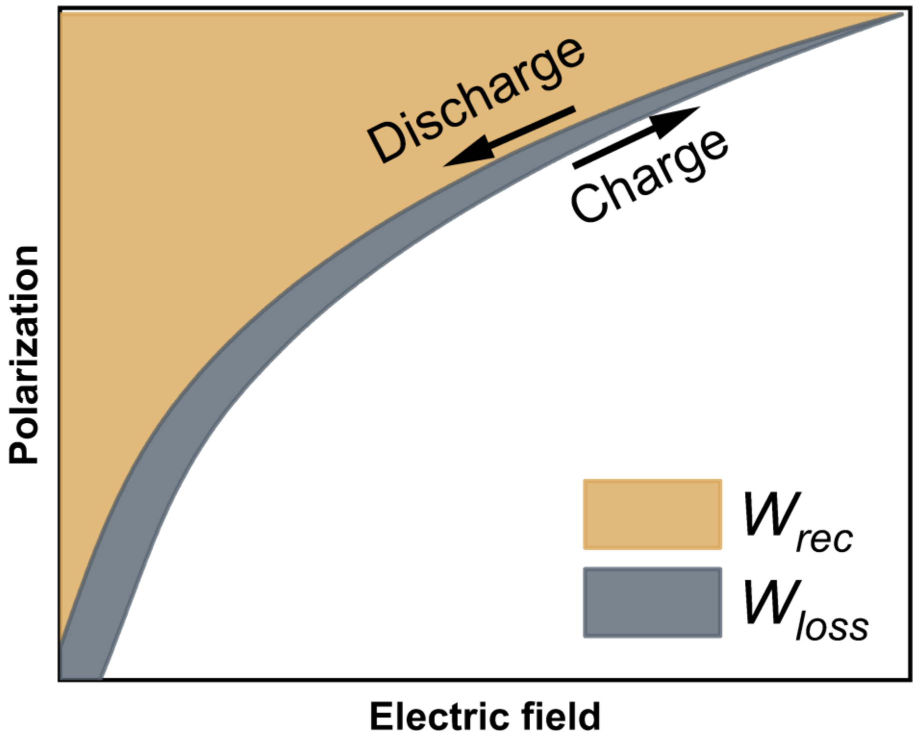

Figure S7. Capacitive energy storage. A schematic illustration of a typical *P*–*E* loop of a RFE during the charge/discharge process. The dischargeable (recoverable) energy density $W_{rec} = \int_{P_r}^{P_{max}} E\, dP$ is represented by the golden area, while the grey area represents the energy loss (W_{loss}). Energy storage efficiency η of the dielectric is defined as $\eta\ (\%) = \frac{W_{rec}}{W_{rec}+W_{loss}} \times 100$.

5. Dielectric breakdown strength

The characteristic breakdown strength E_b was analyzed using two-parameter Weibull distribution described by the formula: $X_i = \ln E_i$, $Y_i = \ln(-\ln(1-p_i))$, $p_i = \frac{i}{n+1}$. Where E_i, i, n, and p_i represent the tested breakdown field of each specimen, the serial number of the tested specimen, the sum of the tested specimens, and the cumulative probability of dielectric failure. E_b is defined as the characteristic breakdown strength, which corresponds to the breakdown field at 63.2% probability of breakdown. Here, 15 breakdown field data points were collected. The breakdown strength of PMN–30PT and Sm-PMN–30PT thin films were obtained from Weibull distribution analysis (Figure S8). Almost double E_b value was obtained in Sm-PMN–30PT film compared to the pure PMN–30PT one. The Weibull modulus β also increases from 3.6 to 13.3 in PMN–30PT and Sm-PMN–30PT film, respectively, indicating narrowed distributions of E_b data and improved film uniformity.[23,24] The substantial enhancement of the breakdown performance is directly ascribed to the prevention of electric and thermal breakdown of the films with the leakage current suppressed by more than two orders of magnitudes (Figure S5d). Besides, we could also attribute the reduction of leakage current and improvement of E_b to the suppression of charge emission by Sm doping, with probable reduction of oxygen vacancies and formation of deep-level defect complexes.[24]

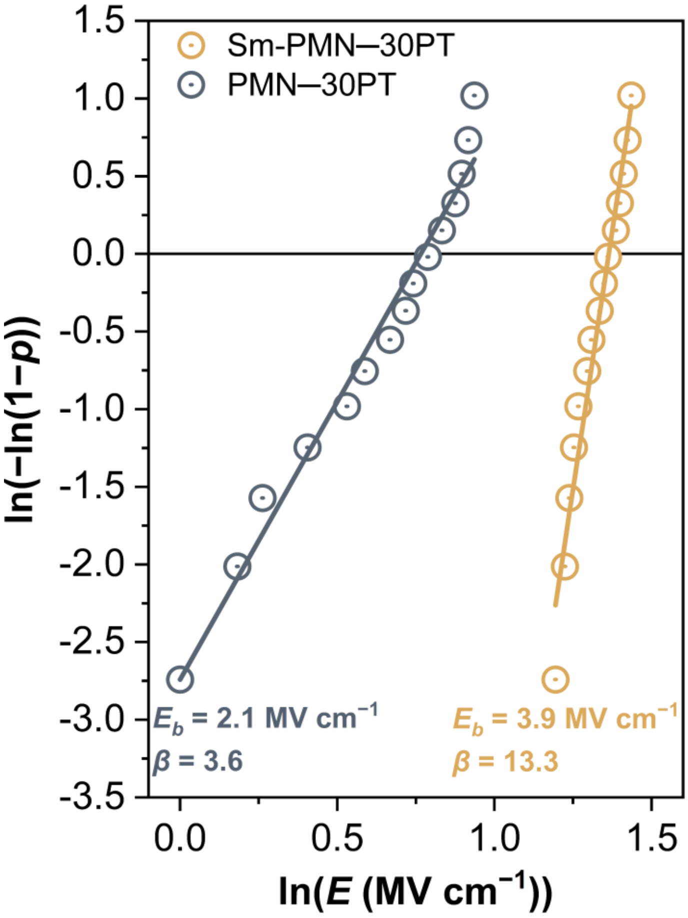

Figure S8. The Weibull distribution of the dielectric breakdown strength of PMN–30PT and Sm-PMN–30PT thin films.

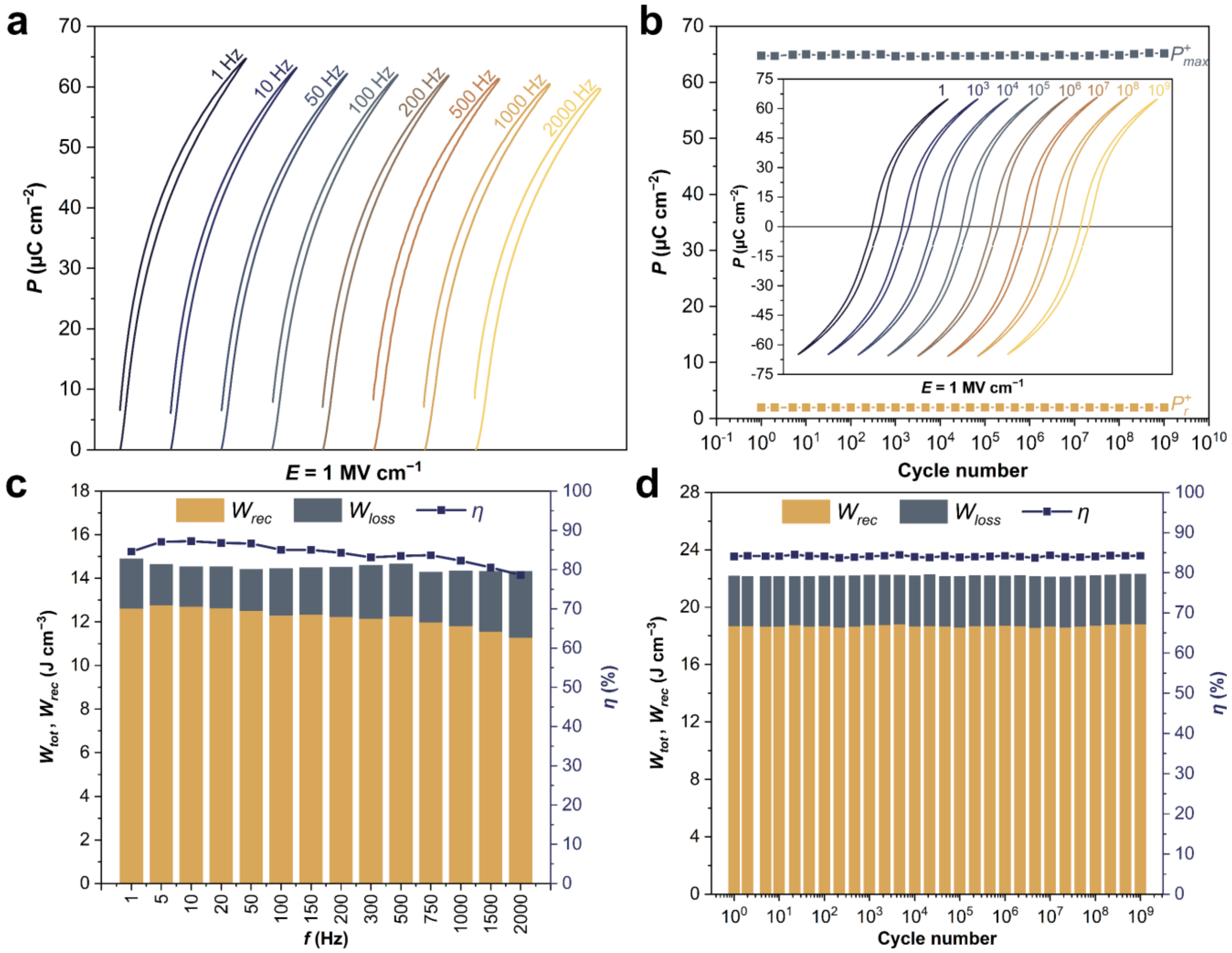

Figure S9. Energy storage stability of Sm-PMN–30PT thin film. (a) Room-temperature unipolar *P*–*E* hysteresis loops recorded at various frequencies at 1 MV cm^{-1}, (b) variation of the polarization at the fatigue of 10^9 cycles (the inset depicts the *P*–*E* shape during cycling) at room temperature at 1 MV cm^{-1}, and (c, d) the corresponding energy storage parameters, respectively, of Sm-PMN–30PT thin film.

6. Electrocaloric and pyroelectric effects

The isothermal entropy change (ΔS) and the electrocaloric temperature change (ΔT) of Sm-PMN–30PT were estimated from the recorded *P–E* loops from 0 to 160°C using Maxwell relations: $\Delta S = -\frac{1}{\rho}\int_{E_1}^{E_2}(\frac{\partial P}{\partial T})_E dE$ and $\Delta T = -\frac{1}{\rho}\int_{E_1}^{E_2}\frac{T}{C_p}(\frac{\partial P}{\partial T})_E dE$. Here, ρ = 8.03 g cm^{-3} and C_p = 320 J kg^{-1} K^{-1} are the mass density (theoretical value) and the specific heat of the sample, respectively.[25] While the pyroelectric effect can be obtained indirectly from *P–E* loops or directly by running thermodynamic cycles, such as Olsen. These indirect methods, based on *P–E* loops collected at different temperatures, were proven to have similar values as the direct approach.[26] Accordingly, we can accurately estimate how much energy can be harvested by calculating the area between the two *P–E* loops at two different temperatures using the formula $N_d = \oint E\, dP$. We have calculated other important parameters like the efficiency's figure of merit $\eta = \frac{N_d}{Q_{in}}$, defined as the ratio of harvested electric energy density N_d over input heat density Q_{in}, Carnot efficiency $\eta_{carnot} = \frac{\Delta T_{span}}{T_{hot}}$, and the relative efficiency (or scaled efficiency) $\eta_r = \frac{\eta}{\eta_{carnot}} = \frac{N_d T_{hot}}{Q_{in}\Delta T_{span}}$ with respect to Carnot. Where $Q_{in} = \rho\int_{T_L}^{T_H} C_p\, dT + Q_{ECE}$ and $Q_{ECE} = \rho C_p \Delta T$ (electrocaloric work). Since the vibrational entropy change (associated with the heat capacity) is typically much larger than the dipolar entropy change, the electrocaloric work can be ignored.[27]

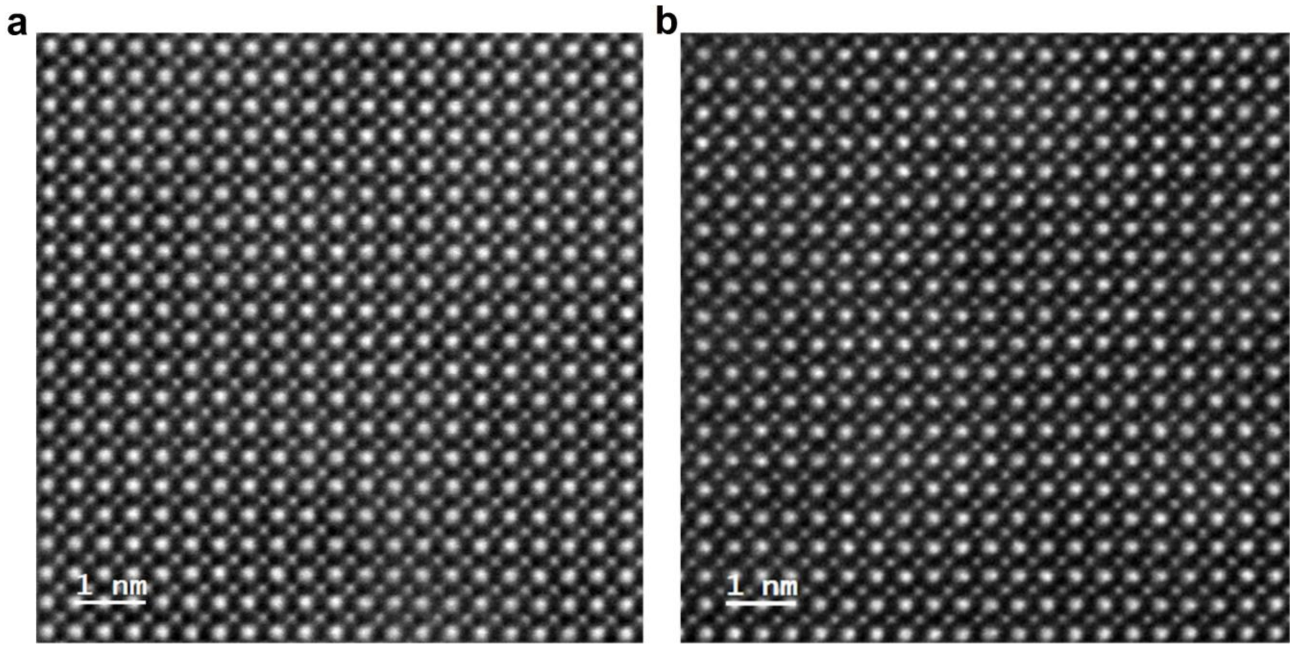

Figure S10. Atomic resolution HAADF-STEM images of a 20 × 20 unit-cell area viewed along the $[100]_{pc}$ zone axis of (a) PMN–30PT and (b) Sm-PMN–30PT thin films. The larger spots correspond to the A-site columns and smaller spots to the B-site columns.

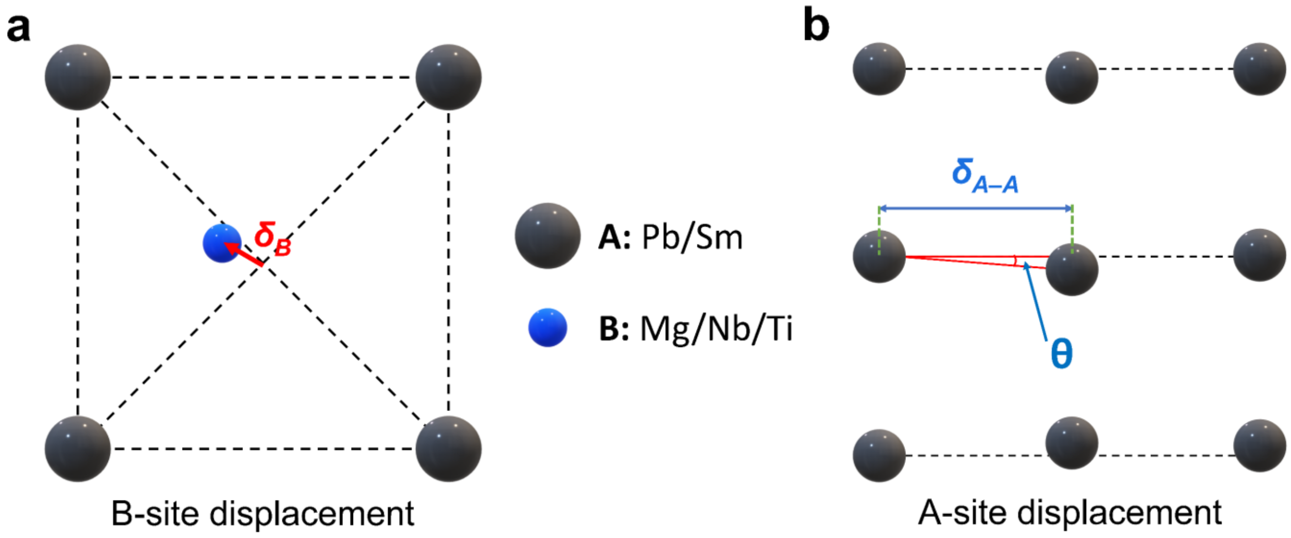

Figure S11. Schematic presentation of the (a) B-site and (b) A-site atoms' displacements.

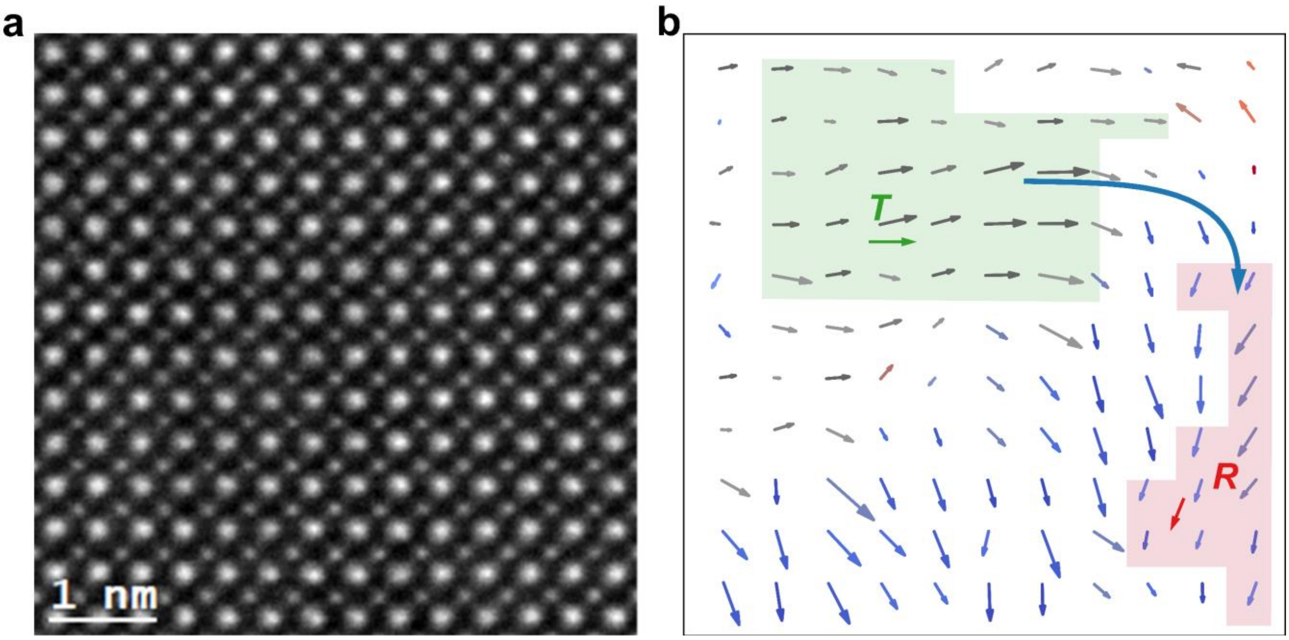

Figure S12. Demonstration of the polymorphic nanodomain structure in Sm-PMN–30PT thin films. (a) Atomic resolution HAADF-STEM images of a 13 × 13 unit-cell area viewed along the $[100]_{pc}$ zone axis. (b) The arrows represent B-site atoms displacement vectors in each unit cell. The red and green colored-plots are the *R* and *T* nanodomains, respectively.

7. A-site positional disorder and local degree of tetragonality

The A-sublattice positional disorder in both samples was evaluated using STEM by calculating the distances between A-site atoms ($\delta_{A\text{–}A}$) on a per-unit cell basis (Figure S13). Although the effective lattice parameters were similar for both samples at approximately 403 pm, the Sm-PMN–30PT displayed a higher standard deviation (SD) of $\delta_{A\text{–}A}$ (10.1 pm) compared to the PMN–30PT film (8.0 pm). This suggests a significantly larger local fluctuation in the Sm-PMN–30PT film. Furthermore, we analyzed the distortion angles of A-site atoms, denoted as θ, which reflects the localized nature of A-atoms off-center displacements, as schematized in Figure S11b. This disorder is observed in both samples, however, the largest local A-site distortions with highest θ angles are observed in Sm-PMN–30PT film. This can be reflected by the large number of brighter islands (Figure S14b), the broader distortion-angle distribution curve, the average distortion angle and its lateral fluctuation (Figure S14d). These strong local A-site distortions are hypothesized to play a key role in the short-range ordering and thus the relaxor behavior of the RFE compositions.[28] Moreover, we assessed the degree of tetragonality $c/a = \frac{c_1+c_2}{a_1+a_2}$ at the A-site positions (Figure S15) and observed higher and more fluctuated c/a values in Sm-PMN–30PT (1.014 ± 0.030) than in the PMN–30PT film (1.005 ± 0.022). These results are consistent with XRD data, suggesting an average rhombohedral structure for PMN–30PT and a more tetragonal structure for Sm-PMN–30PT.

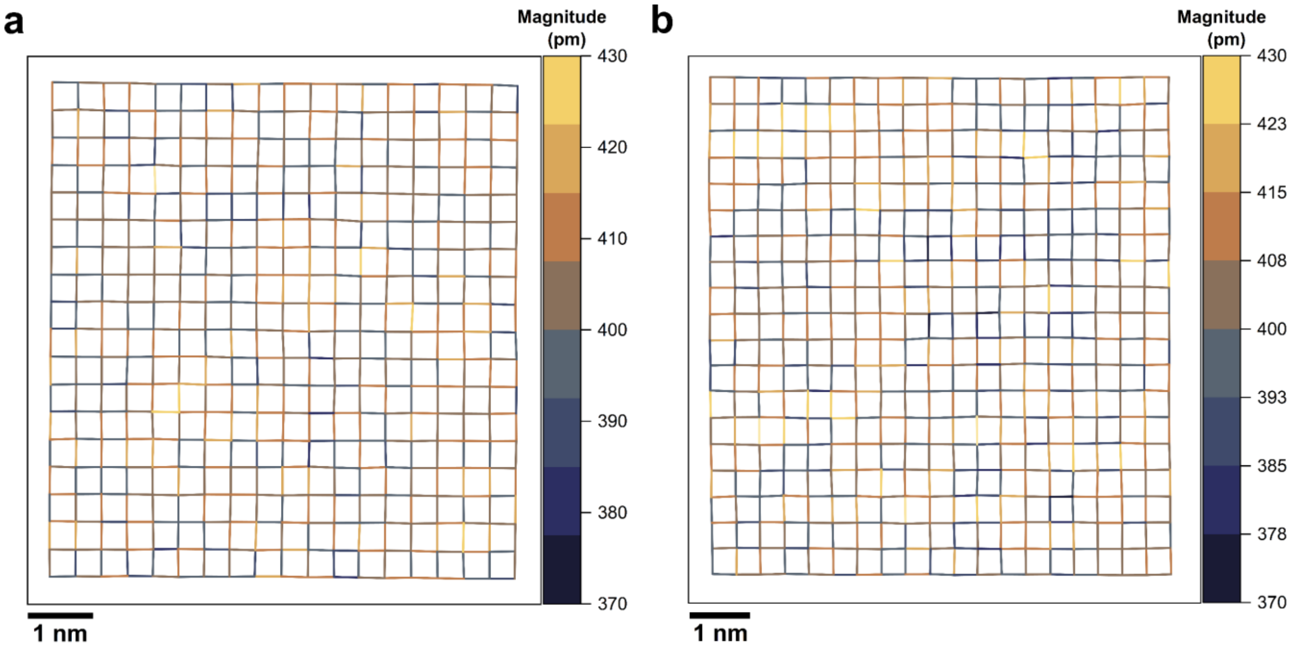

Figure S13. A-site positional disorder maps represented by the distance between A-site atoms for (a) PMN–30PT and (b) Sm-PMN–30PT films.

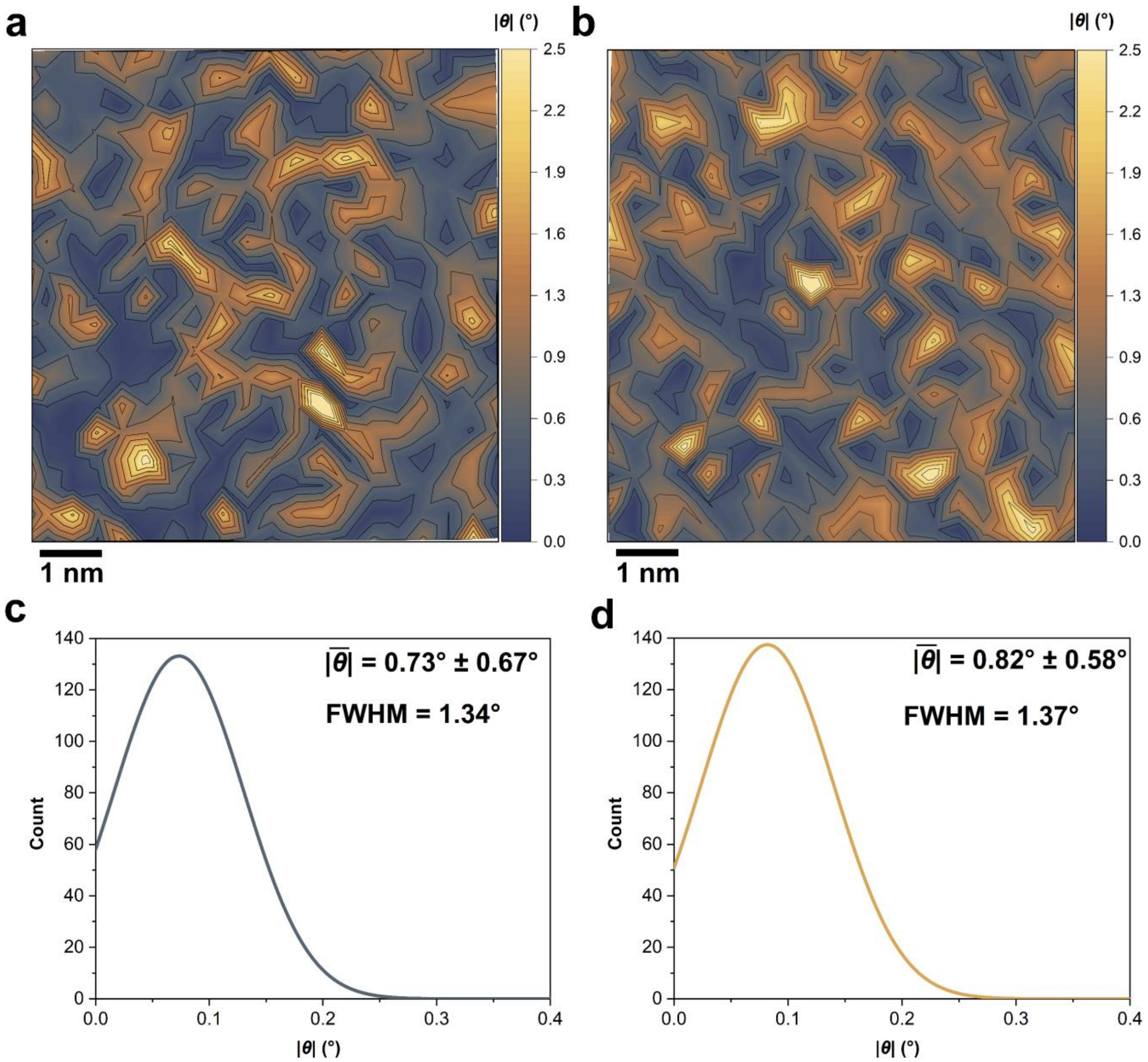

Figure S14. A-sublattice positional disorder maps (with contour lines) for (a) PMN–30PT and (b) Sm-PMN–30PT films, represented by the distortion angle θ, marking the positional deviation of each next A-site atom away from the equatorial line of its neighboring A-site atom (see the schematic on the top-right side in Figure S11b). (c, d) The normal distribution curves of the absolute values of θ with the mean distortion-angle values and standard deviations for (c) PMN–30PT and (d) Sm-PMN–30PT films.

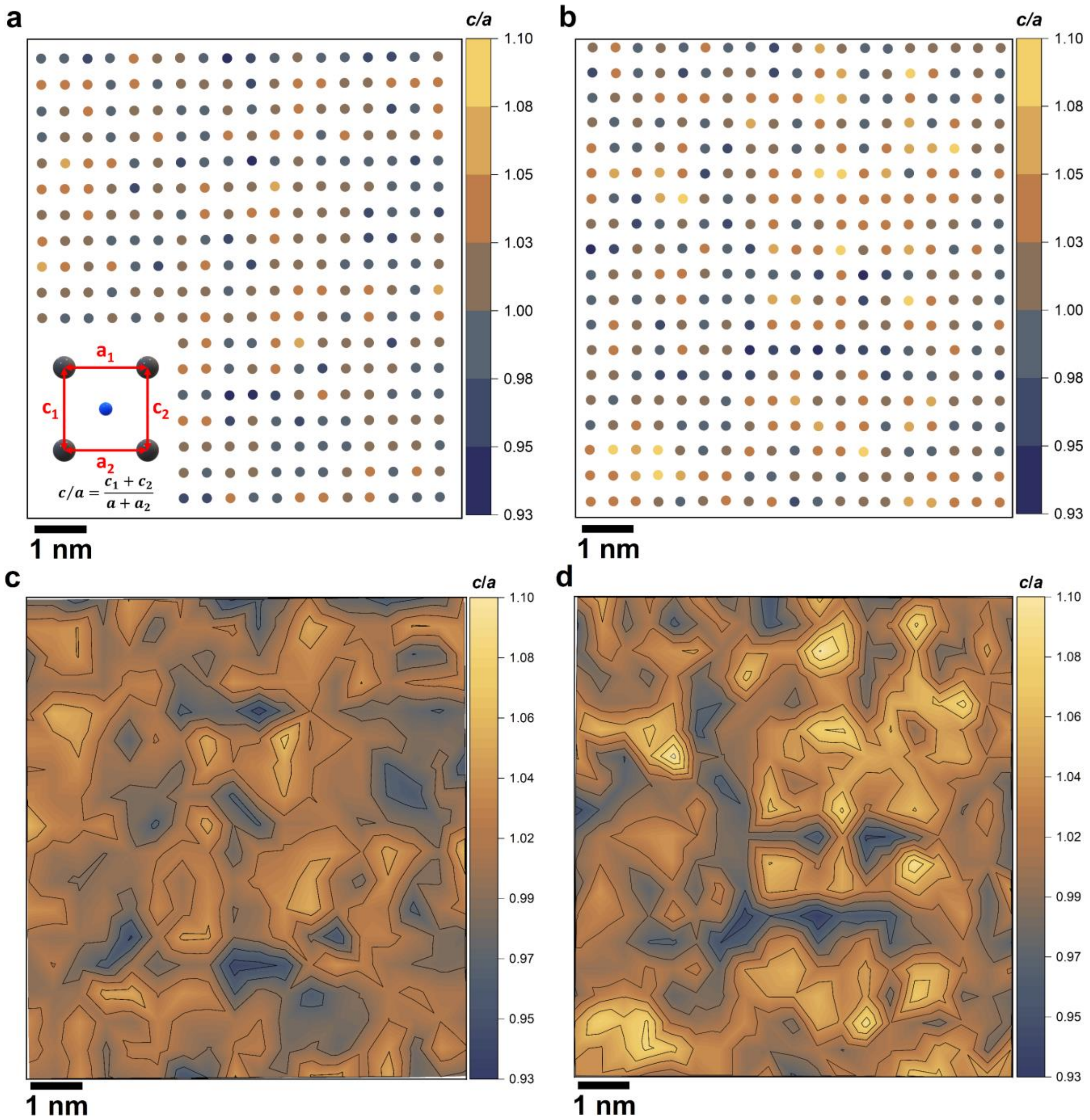

Figure S15. Local degree of tetragonality (*c*/*a*) maps and the corresponding contour lines maps for (a, c) PMN–30PT and (b, d) Sm-PMN–30PT films.

Table S1. Comparison of typical performance parameters in some of the-state-of-the-art electrocaloric materials.

**Material**	***T* (°C)**	***ΔT* (K)**	***ΔS* (J kg^{-1} K^{-1})**	***E* (kV cm^{-1})**	***ΔT×ΔS* (kJ kg^{-1})**	***Q* (kJ kg^{-1})**	**Ref.**
Sm-PMN–30PT	107	59.4	50.1	2000	2.98	19.04	This work
PMN–33PT	152	14.5	10.9	600	0.16	4.64	[29]
PMN–35PT	140	31	32.0	747	0.99	13.22	[30]
PMN–10PT	75	5	5.3	895	0.03	1.84	[31]
PMN–7PT	27	12.2	13.1	723	0.16	3.92	[32]
PMN–7PT	25	9	11.0	723	0.10	3.28	[33]
PZT	25	14.8	11.8	826	0.17	4.87	[34]
PZT	180	17.9	13.1	930	0.23	5.93	[35]
PZT	226	12	8.0	480	0.10	4.0	[36]
PBZ	17	45	47.0	598	2.12	13.63	[37]
PLZST	175	13	9.6	900	0.12	4.29	[38]
PLZST	156	11	8.5	900	0.09	3.63	[39]
PLZST	185	8	5.8	900	0.05	2.64	[39]
PLZT8/65/35	45	40	50.0	1200	2.00	15.90	[40]
$Hf_{0.2}Zr_{0.8}O_2$	34	13.4	16.7	3260	0.22	5.13	[41]
Y-HfO_2	86	24.8	21.7	3500	0.54	7.78	[42]
P(VDF–TrFE)	80	12.5	60.0	2090	0.75	21.18	[43]
P(VDF–TrFE)	67	12	70.0	1200	0.84	23.80	[44]
P(VDF–TrFE)	117	21	87.0	3000	1.83	33.93	[45]
P(VDF–TrFE)	50	28	130.0	1800	3.64	42.0	[46]
P(VDF–TrFE)	33	20	95.0	1600	1.90	29.07	[40]
P(VDF–TrFE–CFE)	55	12.5	64.0	3070	0.80	21.0	[43]
P(VDF–TrFE–CFE)	45	12	55.0	1700	0.66	17.49	[47]
P(VDF–TrFE–CFE)	77	22	85.0	3500	1.87	29.75	[45]

Table S2. Comparison of typical performance parameters in some of the-state-of-the-art pyroelectric materials.

**Material**	***ΔT* (K)**	***Q_{ECE}* (J cm^{-3})**	***E* (kV cm^{-1})**	***N_d* (J cm^{-3})**	***η_r* (%)**	**Ref**
Sm-PMN–30PT	59.4	152.6	2000	40	85.6	This work
PMN–10PT	5	15.0	900	0.4	33.3	31
PMN–32PT	13.4	35.4	600	8	57.3	48
PLZST	20.7	59.4	1092	6.1	67.4	49
PLZST	11	30.1	900	6.8	52.4	39
PLZST	7	19.2	900	4	41.2	39
PLZST	8	21.9	900	3.6	44.4	39
PZT	12	30.1	780	0.6	54.5	36
PST	3.1	-	155	4.4	40.0	26
$Hf_{0.2}Zr_{0.8}O_2$	13.4	30.6	3260	5.7	57.3	41
$Hf_{0.3}Zr_{0.7}O_2$	8.9	21.4	3260	11.5	47.1	41
Si:HfO_2	9.5	36.5	3330	20.3	48.7	50
PVDF–TrFE	12.5	37.6	2000	1.2	55.6	51
P(VDF–TrFE–CFE)	21.6	61.2	3500	1.7	68.4	51

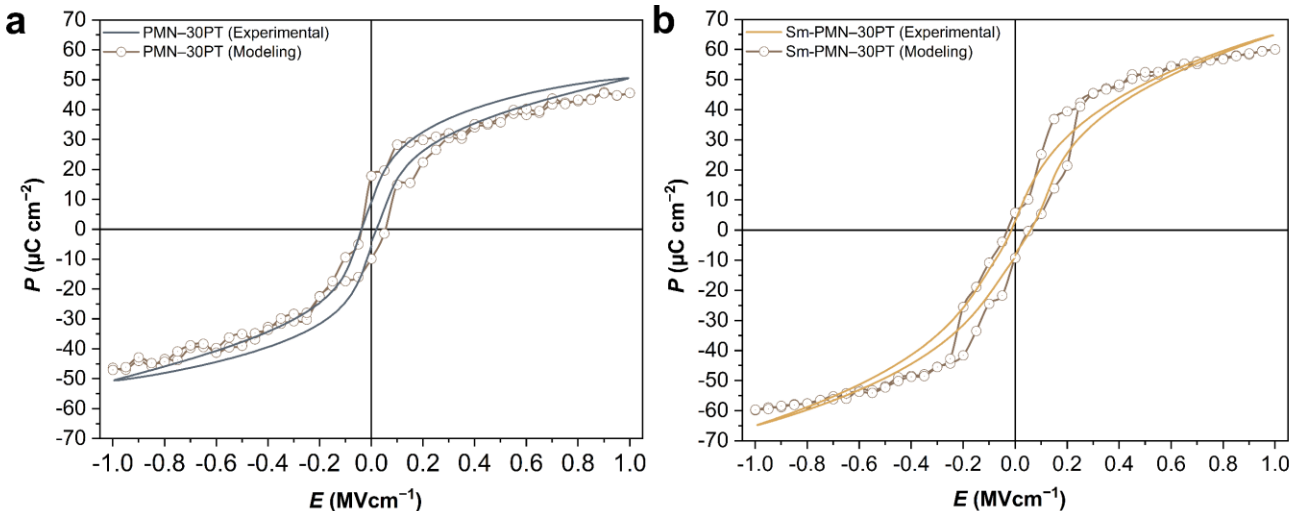

Figure S16. Experimental and phase-field modeled *P*–*E* hysteresis loops at 1 MV cm^{-1} of (a) PMN–30PT and (b) Sm-PMN–30PT films.

References

(1) Nečas, D.; Klapetek, P. Gwyddion: An Open-Source Software for SPM Data Analysis. *Cent. Eur. J. Phys.* **2012**, *10* (1), 181–188. https://doi.org/10.2478/s11534-011-0096-2.

(2) Dycus, J. H.; Harris, J. S.; Sang, X.; Fancher, C. M.; Findlay, S. D.; Oni, A. A.; Chan, T. T. E.; Koch, C. C.; Jones, J. L.; Allen, L. J.; Irving, D. L.; Le Beau, J. M. Accurate Nanoscale Crystallography in Real-Space Using Scanning Transmission Electron Microscopy. *Microsc. Microanal.* **2015**, *21* (4), 946–952. https://doi.org/10.1017/S1431927615013732.

(3) Prosandeev, S.; Wang, D.; Bellaiche, L. Properties of Epitaxial Films Made of Relaxor Ferroelectrics. *Phys. Rev. Lett.* **2013**, *111* (24), 247602. https://doi.org/10.1103/PhysRevLett.111.247602.

(4) Uršič, H.; Prah, U. Investigations of Ferroelectric Polycrystalline Bulks and Thick Films Using Piezoresponse Force Microscopy. *Proc. R. Soc. A Math. Phys. Eng. Sci.* **2019**, *475* (2223), 20180782. https://doi.org/10.1098/rspa.2018.0782.

(5) Haun, M. J.; Furman, E.; Jang, S. J.; McKinstry, H. A.; Cross, L. E. Thermodynamic Theory of PbTiO3. *J. Appl. Phys.* **1987**, *62* (8), 3331–3338. https://doi.org/10.1063/1.339293.

(6) Baudry, L.; Lukyanchuk, I.; Vinokur, V. M. Ferroelectric Symmetry-Protected Multibit Memory Cell. *Sci. Rep.* **2017**, *7* (1), 42196. https://doi.org/10.1038/srep42196.

(7) Wang, J.; Shi, S. Q.; Chen, L. Q.; Li, Y.; Zhang, T. Y. Phase-Field Simulations of Ferroelectric/Ferroelastic Polarization Switching. *Acta Mater.* **2004**, *52* (3), 749–764. https://doi.org/10.1016/j.actamat.2003.10.011.

(8) Luk'Yanchuk, I. A.; Lahoche, L.; Sené, A. Universal Properties of Ferroelectric Domains. *Phys. Rev. Lett.* **2009**, *102* (14), 147601. https://doi.org/10.1103/PhysRevLett.102.147601.

(9) Mokrý, P.; Sluka, T. Identification of Defect Distribution at Ferroelectric Domain Walls from Evolution of Nonlinear Dielectric Response during the Aging Process. *Phys. Rev. B* **2016**, *93* (6), 064114. https://doi.org/10.1103/PhysRevB.93.064114.

(10) Yang, S.; Li, J.; Liu, Y.; Wang, M.; Qiao, L.; Gao, X.; Chang, Y.; Du, H.; Xu, Z.; Zhang, S.; Li, F. Textured Ferroelectric Ceramics with High Electromechanical Coupling Factors over a Broad Temperature Range. *Nat. Commun.* **2021**, *12* (1), 1414. https://doi.org/10.1038/s41467-021-21673-8.

(11) Devonshire, A. F. XCVI. Theory of Barium Titanate. *London, Edinburgh, Dublin Philos. Mag. J. Sci.* **1949**, *40* (309), 1040–1063. https://doi.org/10.1080/14786444908561372.

(12) Logg, A.; Mardal, K. A.; Wells, G. N. *Automated Solution of Differential Equations by the Finite Element Method*; Logg, A., Mardal, K.-A., Wells, G., Eds.; Lecture Notes in Computational Science and Engineering; Springer Berlin Heidelberg: Berlin, Heidelberg, 2012; Vol. 84 LNCSE. https://doi.org/10.1007/978-3-642-23099-8_1.

(13) Geuzaine, C.; Remacle, J. F. Gmsh: A 3-D Finite Element Mesh Generator with Built-in Pre- and Post-Processing Facilities. *Int. J. Numer. Methods Eng.* **2009**, *79* (11), 1309–1331. https://doi.org/10.1002/nme.2579.

(14) Jannelli, A.; Fazio, R. Adaptive Stiff Solvers at Low Accuracy and Complexity. *J. Comput. Appl. Math.* **2006**, *191* (2), 246–258. https://doi.org/10.1016/j.cam.2005.06.041.

(15) Li, F.; Cabral, M. J.; Xu, B.; Cheng, Z.; Dickey, E. C.; LeBeau, J. M.; Wang, J.; Luo, J.; Taylor, S.; Hackenberger, W.; Bellaiche, L.; Xu, Z.; Chen, L. Q.; Shrout, T. R.; Zhang, S. Giant Piezoelectricity of Sm-Doped Pb(Mg1/3Nb2/3)O3-PbTiO3 Single Crystals. *Science (80-.).* **2019**, *364* (6437), 264–268. https://doi.org/10.1126/science.aaw2781.

(16) Prah, U.; Dragomir, M.; Rojac, T.; Benčan, A.; Broughton, R.; Chung, C.-C.; Jones, J. L.; Sherbondy, R.; Brennecka, G.; Uršič, H. Strengthened Relaxor Behavior in (1− x)Pb(Fe 0.5 Nb 0.5)O 3 – x BiFeO 3. *J. Mater. Chem. C* **2020**, *8* (10), 3452–3462. https://doi.org/10.1039/C9TC05883D.

(17) Shvartsman, V. V.; Kholkin, A. L.; Orlova, A.; Kiselev, D.; Bogomolov, A. A.; Sternberg, A. Polar Nanodomains and Local Ferroelectric Phenomena in Relaxor Lead Lanthanum Zirconate Titanate Ceramics. *Appl. Phys. Lett.* **2005**, *86* (20), 1–3. https://doi.org/10.1063/1.1923756.

(18) Rodriguez, B. J.; Gruverman, A.; Kingon, A. I.; Nemanich, R. J.; Cross, J. S. Investigation of the Mechanism of Polarization Switching in Ferroelectric Capacitors by Three-Dimensional Piezoresponse Force Microscopy. *Appl. Phys. A Mater. Sci. Process.* **2005**, *80* (1), 99–103. https://doi.org/10.1007/s00339-004-2925-2.

(19) Shen, B. Z.; Li, Y.; Hao, X. Multifunctional All-Inorganic Flexible Capacitor for Energy Storage and Electrocaloric Refrigeration over a Broad Temperature Range Based on PLZT 9/65/35 Thick Films. *ACS Appl. Mater. Interfaces* **2019**, *11* (37), 34117–34127. https://doi.org/10.1021/acsami.9b12353.

(20) Belhadi, J.; Hanani, Z.; Trstenjak, U.; Shepelin, N. A.; Bobnar, V.; Koster, G.; Hlinka, J.; Pergolesi, D.; Lippert, T.; El Marssi, M.; Spreitzer, M. Large Imprint in Epitaxial 0.67Pb(Mg1/3Nb2/3)O3-0.33PbTiO3thin Films for Piezoelectric Energy Harvesting Applications. *Appl. Phys. Lett.* **2022**, *121* (18), 182903. https://doi.org/10.1063/5.0115777.

(21) Yang, Y.; Sun, E.; Xu, Z.; Zheng, H.; Yang, B.; Zhang, R.; Cao, W. Sm and Mn Co-Doped PMN-PT Piezoelectric Ceramics: Defect Engineering Strategy to Achieve Large D33 and High Qm. *J. Mater. Sci. Technol.* **2023**, *137*, 143–151. https://doi.org/10.1016/j.jmst.2022.08.004.

(22) Li, Y.; Borbely, M.; Bell, A. The Influence of Oxygen Vacancies on Piezoelectricity in Samarium-Doped Pb(Mg1/3Nb2/3)O3-PbTiO3 Ceramics. *J. Am. Ceram. Soc.* **2021**, *104* (6),

2678–2688. https://doi.org/10.1111/jace.17619.

(23) Palneedi, H.; Peddigari, M.; Hwang, G. T.; Jeong, D. Y.; Ryu, J. High-Performance Dielectric Ceramic Films for Energy Storage Capacitors: Progress and Outlook. *Adv. Funct. Mater.* **2018**, *28* (42), 1803665. https://doi.org/10.1002/adfm.201803665.

(24) Pan, H.; Lan, S.; Xu, S.; Zhang, Q.; Yao, H.; Liu, Y.; Meng, F.; Guo, E. J.; Gu, L.; Yi, D.; Wang, X. R.; Huang, H.; MacManus-Driscoll, J. L.; Chen, L. Q.; Jin, K. J.; Nan, C. W.; Lin, Y. H. Ultrahigh Energy Storage in Superparaelectric Relaxor Ferroelectrics. *Science (80-.).* **2021**, *374* (6563), 100–104. https://doi.org/10.1126/science.abi7687.

(25) Goupil, F. Le; Berenov, A.; Axelsson, A. K.; Valant, M.; Alford, N. M. N. Direct and Indirect Electrocaloric Measurements on 〈001〉 - PbMg1/3Nb2/3O3-30PbTiO3 Single Crystals. *J. Appl. Phys.* **2012**, *111* (12), 124109. https://doi.org/10.1063/1.4730338.

(26) Lheritier, P.; Torelló, A.; Usui, T.; Nouchokgwe, Y.; Aravindhan, A.; Li, J.; Prah, U.; Kovacova, V.; Bouton, O.; Hirose, S.; Defay, E. Large Harvested Energy with Non-Linear Pyroelectric Modules. *Nature* **2022**, *609* (7928), 718–721. https://doi.org/10.1038/s41586-022-05069-2.

(27) Pandya, S.; Velarde, G.; Zhang, L.; Wilbur, J. D.; Smith, A.; Hanrahan, B.; Dames, C.; Martin, L. W. New Approach to Waste-Heat Energy Harvesting: Pyroelectric Energy Conversion. *NPG Asia Mater.* **2019**, *11* (1), 26. https://doi.org/10.1038/s41427-019-0125-y.

(28) Otoničar, M.; Bradeško, A.; Fulanović, L.; Kos, T.; Uršič, H.; Benčan, A.; Cabral, M. J.; Henriques, A.; Jones, J. L.; Riemer, L.; Damjanovic, D.; Dražić, G.; Malič, B.; Rojac, T. Connecting the Multiscale Structure with Macroscopic Response of Relaxor Ferroelectrics. *Adv. Funct. Mater.* **2020**, *30* (52), 2006823. https://doi.org/10.1002/adfm.202006823.

(29) Feng, Z.; Shi, D.; Dou, S. Large Electrocaloric Effect in Highly (001)-Oriented 0.67PbMg 1/3Nb2/3O3-0.33PbTiO3 Thin Films. *Solid State Commun.* **2011**, *151* (2), 123–126. https://doi.org/10.1016/j.ssc.2010.11.010.

(30) Saranya, D.; Chaudhuri, A. R.; Parui, J.; Krupanidhi, S. B. Electrocaloric Effect of PMN-PT Thin Films near Morphotropic Phase Boundary. *Bull. Mater. Sci.* **2009**, *32* (3), 259–262. https://doi.org/10.1007/s12034-009-0039-3.

(31) Mischenko, A. S.; Zhang, Q.; Whatmore, R. W.; Scott, J. F.; Mathur, N. D. Giant Electrocaloric Effect in the Thin Film Relaxor Ferroelectric 0.9 Pb Mg1/3Nb2/3O3-0.1 PbTiO3 near Room Temperature. *Appl. Phys. Lett.* **2006**, *89* (24), 242912. https://doi.org/10.1063/1.2405889.

(32) Hamad, M. A. Room Temperature Giant Electrocaloric Properties of Relaxor Ferroelectric 0.93PMN-0.07PT Thin Film. *AIP Adv.* **2013**, *3* (3). https://doi.org/10.1063/1.4795156.

(33) Correia, T. M.; Young, J. S.; Whatmore, R. W.; Scott, J. F.; Mathur, N. D.; Zhang, Q.

Investigation of the Electrocaloric Effect in a PbMg2/3 Nb 1/3 O3 - PbTiO3 Relaxor Thin Film. *Appl. Phys. Lett.* **2009**, *95* (18), 182904. https://doi.org/10.1063/1.3257695.

(34) Chen, C.; Wang, S.; Zhang, T.; Zhang, C.; Chi, Q.; Li, W. Designing Coexisting Multi-Phases in PZT Multilayer Thin Films: An Effective Way to Induce Large Electrocaloric Effect. *RSC Adv.* **2020**, *10* (11), 6603–6608. https://doi.org/10.1039/c9ra10896c.

(35) Zhang, T.; Li, W.; Yu, Y.; Wang, M.; He, J.; Fei, W. Giant Electrocaloric Effect in Compositionally Graded PZT Multilayer Thin Films. *J. Alloys Compd.* **2018**, *731*, 489–495. https://doi.org/10.1016/j.jallcom.2017.10.049.

(36) Mischenko, A. S.; Zhang, Q.; Scott, J. F.; Whatmore, R. W.; Mathur, N. D. Giant Electrocaloric Effect in Thin-Film PbZr0.95Ti 0.05O3. *Science (80-.).* **2006**, *311* (5765), 1270–1271. https://doi.org/10.1126/science.1123811.

(37) Peng, B.; Fan, H.; Zhang, Q. A Giant Electrocaloric Effect in Nanoscale Antiferroelectric and Ferroelectric Phases Coexisting in a Relaxor Pb0.8Ba 0.2ZrO3 Thin Film at Room Temperature. *Adv. Funct. Mater.* **2013**, *23* (23), 2987–2992. https://doi.org/10.1002/adfm.201202525.

(38) Zhao, Y.; Hao, X.; Zhang, Q. A Giant Electrocaloric Effect of a Pb0.97La0.02(Zr0.75Sn0.18Ti0.07)O3 Antiferroelectric Thick Film at Room Temperature. *J. Mater. Chem. C* **2015**, *3* (8), 1694–1699. https://doi.org/10.1039/c4tc02381a.

(39) Hao, X.; Zhao, Y.; Zhang, Q. Phase Structure Tuned Electrocaloric Effect and Pyroelectric Energy Harvesting Performance of (Pb0.97La0.02)(Zr,Sn,Ti)O3 Antiferroelectric Thick Films. *J. Phys. Chem. C* **2015**, *119* (33), 18877–18885. https://doi.org/10.1021/acs.jpcc.5b04178.

(40) Lu, S. G.; Rožič, B.; Zhang, Q. M.; Kutnjak, Z.; Li, X.; Furman, E.; Gorny, L. J.; Lin, M.; Malič, B.; Kosec, M.; Blinc, R.; Pirc, R. Organic and Inorganic Relaxor Ferroelectrics with Giant Electrocaloric Effect. *Appl. Phys. Lett.* **2010**, *97* (16), 162904. https://doi.org/10.1063/1.3501975.

(41) Park, M. H.; Kim, H. J.; Kim, Y. J.; Moon, T.; Kim, K. Do; Hwang, C. S. Toward a Multifunctional Monolithic Device Based on Pyroelectricity and the Electrocaloric Effect of Thin Antiferroelectric HfxZr1-XO2 Films. *Nano Energy* **2015**, *12*, 131–140. https://doi.org/10.1016/j.nanoen.2014.09.025.

(42) Samanta, S.; Anoop, G.; Seol, W. J.; Park, S. M.; Joh, H. J.; Choi, J. O.; Ahn, D.; Unithrattil, S.; Kim, H.; Yeom, J.; Hong, S.; Jo, J. Y. Large Electrocaloric Effect with High Thermal and Electric Field Cycling Stability in Solution-Processed Y:HfO2 Thin Films. *J. Mater. Chem. A* **2022**, *10* (18), 9960–9970. https://doi.org/10.1039/d1ta10229j.

(43) Neese, B.; Chu, B.; Lu, S. G.; Wang, Y.; Furman, E.; Zhang, Q. M. Large Electrocaloric

Effect in Ferroelectric Polymers near Room Temperature. *Science (80-.).* **2008**, *321* (5890), 821–823. https://doi.org/10.1126/science.1159655.

(44) Lu, S. G.; Rožič, B.; Zhang, Q. M.; Kutnjak, Z.; Neese, B. Enhanced Electrocaloric Effect in Ferroelectric Poly(Vinylidene-Fluoride/ Trifluoroethylene) 55/45 Mol % Copolymer at Ferroelectric-Paraelectric Transition. *Appl. Phys. Lett.* **2011**, *98* (12). https://doi.org/10.1063/1.3569953.

(45) Liu, P. F.; Wang, J. L.; Meng, X. J.; Yang, J.; Dkhil, B.; Chu, J. H. Huge Electrocaloric Effect in Langmuir-Blodgett Ferroelectric Polymer Thin Films. *New J. Phys.* **2010**, *12* (2), 023035. https://doi.org/10.1088/1367-2630/12/2/023035.

(46) Li, X.; Qian, X. S.; Gu, H.; Chen, X.; Lu, S. G.; Lin, M.; Bateman, F.; Zhang, Q. M. Giant Electrocaloric Effect in Ferroelectric Poly(Vinylidenefluoride- Trifluoroethylene) Copolymers near a First-Order Ferroelectric Transition. *Appl. Phys. Lett.* **2012**, *101* (13), 132903. https://doi.org/10.1063/1.4756697.

(47) Lu, S. G.; Rožič, B.; Zhang, Q. M.; Kutnjak, Z.; Pirc, R.; Lin, M.; Li, X.; Gorny, L. Comparison of Directly and Indirectly Measured Electrocaloric Effect in Relaxor Ferroelectric Polymers. *Appl. Phys. Lett.* **2010**, *97* (20), 202901. https://doi.org/10.1063/1.3514255.

(48) Vats, G.; Kushwaha, H. S.; Vaish, R.; Madhar, N. A.; Shahabuddin, M.; Parakkandy, J. M.; Batoo, K. M. Giant Energy Harvesting Potential in (100)-Oriented 0.68 PbMg 1/3 Nb 2/3 O 3 –0.32 PbTiO 3 with Pb (Zr 0.3 Ti 0.7) O 3 / PbO x Buffer Layer and (001)-Oriented 0.67 PbMg 1/3 Nb 2/3 O 3 –0.33 PbTiO 3 Thin Films. *J. Adv. Dielectr.* **2014**, *04* (04), 1450029. https://doi.org/10.1142/s2010135x14500295.

(49) Peng, B.; Zhang, Q.; Lyu, Y.; Liu, L.; Lou, X.; Shaw, C.; Huang, H.; Wang, Z. Thermal Strain Induced Large Electrocaloric Effect of Relaxor Thin Film on LaNiO3/Pt Composite Electrode with the Coexistence of Nanoscale Antiferroelectric and Ferroelectric Phases in a Broad Temperature Range. *Nano Energy* **2018**, *47*, 285–293. https://doi.org/10.1016/j.nanoen.2018.03.003.

(50) Hoffmann, M.; Schroeder, U.; Künneth, C.; Kersch, A.; Starschich, S.; Böttger, U.; Mikolajick, T. Ferroelectric Phase Transitions in Nanoscale HfO2 Films Enable Giant Pyroelectric Energy Conversion and Highly Efficient Supercapacitors. *Nano Energy* **2015**, *18*, 154–164. https://doi.org/10.1016/j.nanoen.2015.10.005.

(51) Defay, E.; Crossley, S.; Kar-Narayan, S.; Moya, X.; Mathur, N. D. The Electrocaloric Efficiency of Ceramic and Polymer Films. *Adv. Mater.* **2013**, *25* (24), 3337–3342. https://doi.org/10.1002/adma.201300606.
